# Time-varying data processing with nonvolatile memristor-based temporal kernel

**DOI:** 10.1038/s41467-021-25925-5

**Published:** 2021-09-30

**Authors:** Yoon Ho Jang, Woohyun Kim, Jihun Kim, Kyung Seok Woo, Hyun Jae Lee, Jeong Woo Jeon, Sung Keun Shim, Janguk Han, Cheol Seong Hwang

**Affiliations:** 1 Department of Materials Science and Engineering College of Engineering, Seoul National University, Seoul, 08826 Republic of Korea; 2 Inter-university Semiconductor Research Center, Seoul National University, Seoul, 08826 Republic of Korea

**Keywords:** Electronic devices, Electronic devices

## Abstract

Recent advances in physical reservoir computing, which is a type of temporal kernel, have made it possible to perform complicated timing-related tasks using a linear classifier. However, the fixed reservoir dynamics in previous studies have limited application fields. In this study, temporal kernel computing was implemented with a physical kernel that consisted of a W/HfO_2_/TiN memristor, a capacitor, and a resistor, in which the kernel dynamics could be arbitrarily controlled by changing the circuit parameters. After the capability of the temporal kernel to identify the static MNIST data was proven, the system was adopted to recognize the sequential data, ultrasound (malignancy of lesions) and electrocardiogram (arrhythmia), that had a significantly different time constant (10^−7^ vs. 1 s). The suggested system feasibly performed the tasks by simply varying the capacitance and resistance. These functionalities demonstrate the high adaptability of the present temporal kernel compared to the previous ones.

## Abstract (web summary)

Recently there has been an interest in utilising memristors as physical temporal kernels. Here, Jang *et al* demonstrate a physical temporal kernel using a memristor combined with a capacitor and resistor, where the additional circuit elements can be varied to allow the system to tackle a diverse range of tasks.

## Introduction

Convolutional neural networks (CNNs), which are composed of a convolutional layer and a fully connected layer^1^, show outstanding performance in static image processing (recognition and classification)^2,3^. However, when the temporal order of each input vector and the correlation between the input vectors are essential, such as for natural language recognition or translation, a method of processing the input overtime is required, and CNNs are not suitable for this purpose^4^. Such an event-sequence or time-dependent network operation can generally be represented by the relationship between the present network state, the input, and the previous network state.

A typical network with such characteristics is a recurrent neural network (RNN) with the long-short-term memory learning rule^5^, which mitigates the vanishing gradient-descent problem of the classical RNN^6^. Nonetheless, these artificial neural networks perform vast amounts of multiplication and accumulation (MAC) operations during the learning and inference steps. When these calculations are performed using the conventional architecture in which the computing unit and memory are separated, even with the latest graphics processing unit, the cost of achieving the required processing speed and the energy consumption are enormous^7^.

In this regard, the recent upsurge of studies on neural networks that use a memristor-based cross-bar array (CBA) based on Ohm’s law and Kirchoff’s law is notable^8–13^. If the memristor used in such neural networks can process the event-sequence-related and temporal information, it can achieve RNN functionality. An even more desirable functionality is to extract the features of the input information (raw data vector) using a temporal kernel (TK) and feed them to the next classification layer. A representative example of such a computing system is reservoir computing (RC), which is composed of a reservoir and a readout layer (FCN)^14,15^.

The core part of the RC system is the reservoir, where the nonlinear transformation of the input signal is performed based on the fading-memory properties, and the characteristics of the input signal are projected into a rich enough feature space. The result of the projection is called the *reservoir state*^16^.

The nonlinear dynamic filtering of RC can be regarded as a specific type of a more general TK^17–19^, in which the time-varying data can be efficiently handled by the fading-memory functionality of the reservoir. Nonetheless, RC may have severe limitations in adapting different time scales of the input data due to its fixed time constant of the specific fading-memory function. This may not be the case for other types of TK, based on a physical kernel combined with other circuit elements, as shown in this work. Also, non-fading (or nonvolatile) memory can be used as the TK because the time-varying input can be encoded into the TK by the effects of the time constant of the entire circuit element. When a memristor is used as the TK, its resistance must be determined by the different input pulse signals with varying amplitudes and the intervals between such input signals. If the input signals have simple and obviously distinguishable patterns, a memristor can sufficiently discern them by assigning different resistance values. However, for complicated and similar input patterns, high separability is required, which is usually challenging to achieve with a given type of memristor^20,21^. Also, the input signals could have substantially different time constants, which further severely limits the memristor-based temporal kernel (reservoir)^22,23^. In this case, a high-performance kernel machine applicable to diverse circumstances can be created by incorporating additional circuit components.

Recently, various studies were conducted on hardware-based RC systems that use volatile memristors, in which a volatile memristor was used to process a time-varying input^20–23^. In those studies, the reservoirs were constructed based on ionic diffusion dynamics (diffusive memristors), in which the spontaneously decaying conductance of low-resistance state (LRS) of the diffusive memristor provided the fading-memory function of a reservoir.

However, there are several limitations in using such reservoir dynamics. Firstly, the duration and interval of the input signal are limited to the time range in which sufficient conductance decay occurs. For this reason, in the previous studies, it took 1–20 ms for one memristor to process 4-bit data, which is insufficient for processing a large amount of data^20,21^. Secondly, obtaining a reproducible reservoir state could be challenging. An Ag-filament-based diffusive memristor exhibits stochastic switching^20^, so the variation of the reservoir state will be large. Finally, reservoir adaptation could be difficult to achieve, given that the reservoir dynamics are totally determined by the material property, which renders the previous system useful only for applications with a time scale similar to that of the specific memristor^21–23^.

In this study, a device based on an electron trap/detrap mechanism was used to solve the aforementioned issues^24,25^. A W/HfO_2_/TiN (WHT) memristor goes into an LRS when the trap is filled with electrons and shifts to a high-resistance state (HRS) when the trapped electrons are detrapped. Since the resistance switching is based on the electron trapping and not the ionic movement, reproducible results can be achieved (Supplementary Fig. S1a–d)^26,27^. In addition, since the work functions between the top and bottom electrodes differ only slightly, there is limited built-in potential, so the device has high retention properties (Supplementary Fig. S2a, b)^25,28^. Although the WHT memristor has different time constants of operation according to its conductance level (Supplementary Fig. S2c), it is insufficient to achieve adaptability with a sufficiently large time constant range. This problem could be solved by combining the memristor with a capacitor (*C*) and a normal resistor (*R*). Under this circumstance, the *R*–*C* time constant of the circuit can be varied, and the memristor response to the temporal arrangement of the inputs can be controlled.

## Results

**Fig. 1:**
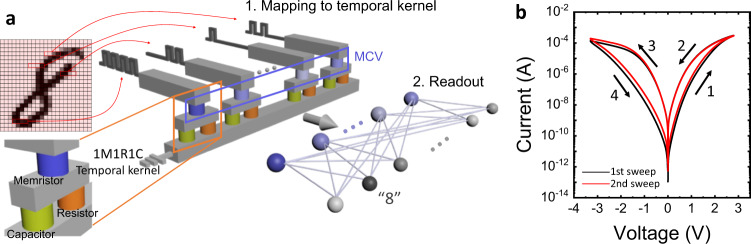
The structure of the 1M1R1C temporal kernel system and the *I–V* characteristics of the memristor used in the temporal kernel. **a** The structure of the 1M1R1C temporal kernel system proposed in this study. The temporal kernel system can recognize images in the MNIST database through feature projection and classification. **b** The *I–V* curve of the W/HfO_2_/TiN memristor. The sweep order is marked in the figure. SET and RESET occurred in the positive bias and the negative bias, respectively, and gradual switching occurred in both switching conditions. Since the filament formation process is not required in this electronic switching device, no electroforming process is seen in the first sweep.

Figure 1a shows the TK system that can control the kernel dynamics using a memristor, a normal resistor, and a capacitor (1M1R1C). This is a structure in which the reservoir is replaced with a 1M1R1C temporal kernel while maintaining the computing scheme of the RC system. In this TK system, the charging and discharging of the capacitor transforms the signals applied to the device into various forms so that the conductance state of the memristor can be varied depending on the magnitude and sequential arrangement of the input signal (Supplementary Fig. S3a, b). The results of input processing in the kernel form a memristor conductance vector (MCV), which becomes the input of the subsequent FCN readout layer. Such a configuration of the TK system allows the arbitrary variation of the response dynamics by adjusting the sizes of the resistor, capacitor, and pulse width, etc. Therefore, the optimized TK system can be configured for tasks with vastly different time scales.

### Device analysis

Figure 1b shows the measured current–voltage (*I–V*) curve of the WHT device. During the electrical measurement, the W top electrode (TE) was biased, while the TiN bottom electrode (BE) was electrically grounded. The resistance of the device was changed from HRS to LRS by a positive bias (SET), and reverse switching was achieved by a negative bias (RESET). In both SET and RESET, gradual switching appeared, as shown in Fig. 1b and Supplementary Figs. S3a, b, which contributed to the high performance of the TK system. Supplementary Fig. S3c shows the cross-sectional scanning transmission electron microscopy (STEM) image of the WHT device, which revealed the W TE, the TiN BE, and the 4 nm thick HfO_2_ layer between the TE and BE. Supplementary Fig. S3d shows the X-ray photoelectron spectroscopy (XPS) analysis of the W/HfO_2_ interface in the WHT device. Analysis of the W peak in the XPS data revealed the presence of tungsten sub-oxide (WO_x_, *x* < 2) and a WO_3_ layer. The energy-dispersive X-ray spectroscopy line scan result (Supplementary Fig. S3c, right portion) along the vertical line from TE to BE in the STEM image implies that a thin WO_3_ was formed at the W/HfO_2_ interface and WO_*x*_ (*x* ≪ 3) was formed within the W bulk. Therefore, the WO_*x*_ may work as a voltage divider when the voltage is applied to the device, which will cause gradual SET and RESET performance^29^. This is a favorable characteristic, allowing the TK to have various states. Moreover, this WHT device does not have an electroforming step (Fig. 1b), which also contributed to the stable resistance switching operation (Supplementary Figs. S4–5 and Supplementary Note 1). W and TiN have similar work functions of ~4.5 eV, which may render the energy band profile symmetric^30,31^. The symmetric energy band profile is unfavorable for fluent electronic bipolar resistive switching (eBRS)^25,28^. However, the WO_3_ layer formed at the W/HfO_2_ interface can induce a Schottky barrier, whereas the HfO_2_/TiN interface constitutes a quasi-ohmic contact^29,32^. Especially, the chemical interaction between the HfO_2_ and TiN layers can produce defects within the HfO_2_ layer, which provide the system with the electron traps that are necessary to induce the eBRS mechanism. With the application of the positive bias to the TE, the traps were filled with electrons that were injected from the TiN BE through the quasi-ohmic contact, which switched the device to the LRS. Conversely, when the negative bias was applied, the device switched back to the HRS as the trapped electrons were detrapped, while the electron injection from the TE was blocked by the Schottky barrier at the W/HfO_2_ interface^28^. Due to the presence of the WO_*x*_ layer, there was no need to set current compliance (CC) during the operation.

### Temporal kernel generation

**Fig. 2:**
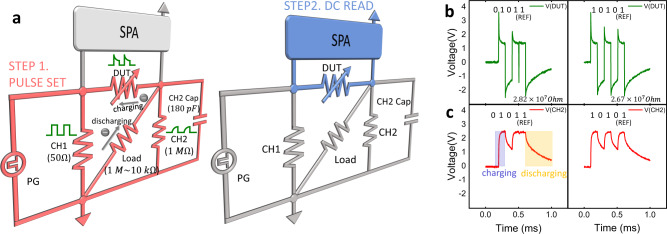
The circuit used as a temporal kernel in the experiment, and the V-t graphs obtained from the DUT and CH2 of this circuit. **a** A temporal kernel circuit composed of a memristor, resistors, and a capacitor. CH1 shows the shape of the input pulse stream, and CH2 shows the voltage applied to a 1 MΩ resistor. The voltage across the DUT (green graph) is obtained by subtracting the CH2 voltage from the CH1 voltage. The left panel shows the circuit used in the pulse set (marked by pink) and the right panel shows the circuit used in DC read (marked by blue). **b** The voltages applied to the memristor with a ‘0101+reference pulse’ (left) and a ‘1010+reference pulse’ (right). **c** The voltages applied to the corresponding CH2, where the 4 V and 0 V voltage amplitudes represent ‘1’ and ‘0,’ respectively. The voltage across CH2 shows that the charging and discharging rates of the capacitor were asymmetric.

We implemented the TK by configuring the circuit, as shown in Fig. 2a. Pulse streams were generated by a pulse generator (PG), where input signal ‘1’ is converted to a high level, and ‘0’ is converted to a low level. These pulse streams were delivered to channel 1 (CH1) and channel 2 (CH2) of an oscilloscope (OSC). A 50 Ω resistor was assigned to CH1, which allowed monitoring of the input pulse shape. In CH2, a 1 MΩ resistor was connected to the device-under-test (DUT, the memristor) in series. From the estimated voltage from the CH2 resistor, the voltage applied to the DUT was inferred. Since the oscilloscope fixes the size of the CH2 resistor at 1 MΩ, the overall series resistance to the memristor was adjusted by connecting a load resistor (*R*_L_), as shown in the figure. Also, a capacitor was connected to the CH2 resistor in parallel, which stored the charge supplied by the applied pulse voltage. In this specific experimental setup, its value was fixed at 180 pF, but the dynamic time constant of the TK system was varied by changing *R*_L_ and the capacitance. The measurement consisted of two steps. In the first step, a pulse was generated at the PG, which caused SET switching in the memristor, while the circuit part with the semiconductor parameter analyzer (SPA) was deactivated (Fig. 2a left panel). In the second step, the conductance state of the memristor was read through the DC sweep using the SPA, while the other parts of the circuit were deactivated (Fig. 2a right panel).

Figure 2b shows the voltages transients over the memristor with a ‘0101+reference pulse’ (left) and a ‘1010+reference pulse’ (right), and Fig. 2c shows the corresponding voltage transients read at CH2. In these operations, 4 V, 200 μs, and 0 V, 200 μs pulses were programmed to represent ‘1’ and ‘0’, respectively. The initial resistance of the WHT memristor was set to 50 MΩ when measured at 0.5 V. The role of the last reference pulse is explained as follows. The left panels of Fig. 2b and c show that since the first signal was ‘0’, no voltage appeared up to 0.2 ms. When the first ‘1’ signal was applied, the DUT showed a peak of up to ~3.5 V due to the involvement of the capacitive charging current, and it decayed to ~1.5 V after the capacitor charging was completed. At the same time, the CH2 voltage showed a corresponding gradual increase in the capacitor voltage, which was saturated at ~2.5 V. When the second ‘0’ signal came in, the capacitor was discharged and the reverse current flowed into the DUT, which made its voltage negative, while the CH2 showed gradual decay of the capacitor voltage. It was noted from the CH2 voltage that the capacitor was not completely discharged during the 0.2 ms duration of ‘0’ signal, so when the subsequent ‘1’ signal came in, the capacitive charging current was not as high as in the previous ‘1’ signal case (where the DUT voltage peaked only up to ~2.5 V). Such an effect can be more evidently seen with the subsequent ‘1’ signal (the reference pulse), as there was almost no peak in the DUT. Therefore, in this case, the effective number of SET pulses applied to the DUT was only two (the first and second ‘1’ among the total three ‘1’s in the ‘01011’ sequence). After the entire pulse sequence was over, the memristor resistance was 28.2 MΩ.

In the case of the right panels in Fig. 2b and c, in contrast, each of the 1 signals is separated by 0 signals, and all the three ‘1’s in the ‘10101’ sequence are effective, and they switched the DUT to the SET state, which made its resistance 26.7 MΩ, despite the application of the same number of set pulses (three) in the two cases. It should be noted, however, that the last two peaks had a lower effect in decreasing the memristor resistance than the first one due to its lower peak height, which was induced by the incomplete discharging of the capacitor during the intervening ‘0’ pulse cycle. This is not a demerit but actually a merit of this TK system, which allowed even higher separability and adaptability. Therefore, this TK system can recognize not only the different input pulse numbers but also their timing. Figure 2b, c shows several notable features. First, due to the built-in asymmetry of the band profile of the WHT memristor, the resistance at the positive bias of ~2.5 V was ~100 times lower than that at the negative bias of ~1.5 V. Therefore, the charging was much faster than the discharging. This is the first factor that allows the TK system to have higher separability and adaptability. Second, the capacitance and *R*_L_ can be arbitrarily taken to vary the charging and discharging times, which can eventually affect the effectiveness of the voltage pulse applied to the memristor. Third, the input voltage pulse height and duration are another knob that can further change the TK dynamics. These features rendered the TK system flexible and adaptable to the various requirements, as shown in the next sections. The reference pulse ‘1’ after the pulse stream is required to recognize the change in the charge level. Without the last reference pulse, such a systematic variation and examination of the memristor state control would have been improbable.

The WHT memristor in this study shows both nonvolatile and volatile memory properties, when its conductance is low and high, respectively. In this study, the WHT memristor was operated within the conductance range showing nonvolatile characteristics, but outside that range, the WHT device shows fading conductance state (Supplementary Fig. S2c). Therefore, depending on the operation scheme, the 1M1R1C kernel can also perform a reservoir function, and the results are shown in Supplementary Fig. S6. In this study, time-series data were processed based on the unique characteristics of 1M1R1C, not the fading memory.

### Modifying the temporal kernel dynamics

**Fig. 3:**
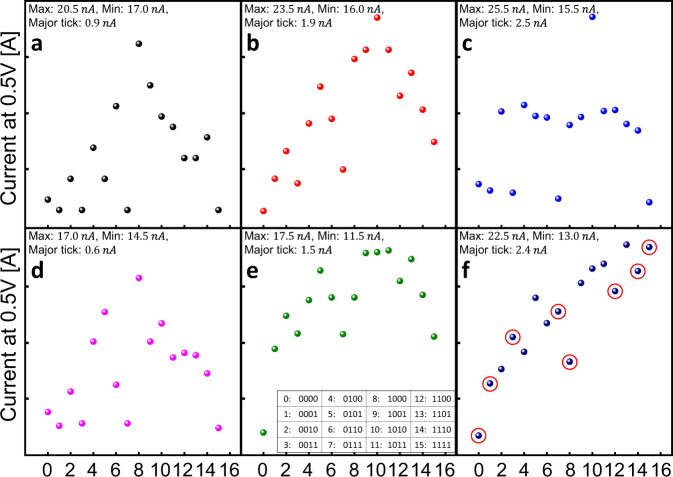
Experiment results to analyze the effect of changing parameters on the kernel characteristics in the temporal kernel system. The read current at 0.5 V of the memristor for the pulse stream ‘0000’–‘1111’ that corresponds to 0–15 in the inset table in (**e**). **a** The read current at 0.5 V of the memristor for each input under the conditions of 1 MΩ R_L_, 4 V signal pulse height, 100 µs width, 4 V REF pulse height, and 100 µs width. **b**–**e** The read current at 0.5 V of the memristor for each input when R_L_, pulse width, pulse height, and REF pulse height are changed respectively from the condition of (**a**). The various parameter settings for each figure were summarized in Table 1. The kernel responses for each input of the temporal kernel optimized for the MNIST recognition are shown in (**f**). Responses to inputs showing high prevalence in the dataset were well separated (marked by red circles).

In this TK system with the given WHT memristor property and capacitance, R_L_ and the pulse height/duration were varied to examine the separability of the memristor. The capacitance could also be varied, but it was fixed in this experiment section. Figure 3 shows several examples of the different degrees of separability of the TK system when these parameters were varied. The examples show the current value read at 0.5 V after the 16 different input patterns, from ‘0000’ to ‘1111’, were programmed to the PG, with the additional reference pulse added last. Since the output current depends on the initial resistance, the resistance of the WHT memristor in this experiment was reset to a constant value (50 MΩ at 0.5 V) before measurement. The x-axis numbers correspond to the different input patterns described in the inset table in Fig. 3e, and the different parameters, such as R_L_, the input pulse, and the reference pulse, for each graph in Fig. 3 are summarized in Table 1. It should be noted that in Fig. 3, the *y*-axis scales of each graph were varied to easily compare them. All the detailed pulse responses and analyses are included in Supplementary Figs. S7–11 and Supplementary Note 2. In Fig. 3a, wherein *R*_L_ = 1 MΩ, the signal pulse = 4 V, 100 µs, and the reference pulse = 4 V, 100 µs, the five patterns, ‘0000’, ‘0001’, ‘0011’, ‘0111’, and ‘1111’ are not clearly distinguished (an analysis of the separation of these inputs is shown in Supplementary Fig. S12). It was also noted that the ‘1000’ pattern resulted in the highest memristor conductance, although there were only two SET pulses (the first 1 and the reference pulse at the last SET pulse). This is because the reference pulse induced the highest peak voltage to the memristor because the interval between the two pulses, during which the capacitor was fully discharged, was the longest (the details are shown in Supplementary Fig. S13).

**Table 1:** The temporal kernel conditions (RL, signal pulse, and REF pulse) used in Fig. 3a–e.

TK condition	*R*_L_	Signal pulse	REF pulse
Fig. 3a	1 MΩ	4 V, 100 μs	4 V, 100 μs
Fig. 3b	120 kΩ	4 V, 100 μs	4 V, 100 μs
Fig. 3c	1 MΩ	4 V, 200 μs	4 V, 100 μs
Fig. 3d	1 MΩ	3.5 V, 100 μs	3.5 V, 100 μs
Fig. 3e	1 MΩ	4 V, 100 μs	3 V, 100 μs
Fig. 3f	10 kΩ	3.5 V, 200 ns	3 V, 200 ns

Of the six graphs in Fig. 3, Fig. 3c shows well the critical features of this TK system. The only difference of Fig. 3c from Fig. 3a is the pulse length [100 μs (in a) vs. 200 μs (in c)]. As the pulse width increases, the capacitor discharging during the 0 input increased, and the subsequent ‘1’ induced a higher peak voltage. The conductance levels in Fig. 3c can be clearly grouped into three levels, which are determined by the number of 1’s immediately after the ‘0’ (not the total number of ‘1’). For example, ‘0000’ has only one 1 after 0 (the reference pulse), so it induced the lowest conductance. Interestingly, ‘1111’ has the same low conductance even though it had five 1 inputs (including the reference pulse). This is because the only effective ‘1’ was the first one because all the other ‘1’s do not have the preceding ‘0’s, so they cannot produce peak voltage.

Another characteristic and most desirable setting could be seen in Fig. 3f, in which *R*_L_ was decreased to 10  kΩ and the pulse width was decreased to 200 ns. This setting makes the capacitor charging per one voltage pulse (‘1’ signal) insufficient and its discharging during the ‘0’ signals faster. Overall, this makes the memristor conductance more linearly dependent on the total number of ‘1’s, as shown in Fig. 3f (an example of insufficient charging and details of the effects are included in Supplementary Fig. S14). A short pulse length is also beneficial to rapidly process the input vectors.

By appropriately changing both the *C* and *R*_L_, the kernel characteristics obtained in Fig. 3 could be implemented at different time scales. Additional kernels are configured as the time constants in Supplementary Fig. S15. Based on the analysis of the effect of each parameter change, a kernel condition suitable for the task is determined through kernel adaptation, and ex-situ training is performed, which is followed by inference.

### Task optimization: MNIST

To perform the task of recognizing digit images in the Modified National Institute of Standards and Technology (MNIST) Database, the kernel dynamics were optimized to implement a TK system suitable for the task. To do this, the raw MNIST dataset, composed of 784 pixels (28 × 28), had to be reconfigured to meet the requirement of this specific TK system, which is basically a binary system (0 and 1 inputs). Therefore, the data in the 784 pixel images were binarized and chopped by 4 bits, which resulted in 196 4-bit input signals. To make the task analysis more efficient, the frequency of the appearance of inputs in the dataset was investigated, and it was confirmed that ‘0000’ appeared most frequently, followed by ‘1111,’ ‘1000,’ ‘0011,’ and ‘0001’ (Supplementary Table 1). Therefore, in this task-optimized TK system, the task was performed effectively by setting the operation parameters so that the TK system could readily separate the responses to the inputs with a high frequency of appearance rather than separating the responses to all the 16 inputs. The data points indicated by the red circle in Fig. 3f correspond to these frequently appearing signal sets. Accordingly, the 196 4-bit input image data were converted to the 196 membered MCV, where the measurements were performed on a single 1M1R1C circuit, based on Fig. 3f. Using the 50,000 training images in the MNIST dataset, 50,000 training MCVs were generated. These MCVs were used to train the 196 × 10 FCN (weights and biases), which were generated in a PyTorch simulation (Methods section). The trained TK system was used to infer the 10,000 MNIST test images, and the achieved accuracy was 90.1% (see Table 2 and Supplementary Tables 2, 3 for the results of combining various kernels and the results of considering cycle-to-cycle and cell-to-cell variations). When one hidden layer composed of 200 neurons is added to the FCN, the accuracy was increased to 96.5%.

**Table 2:** Comparison of the results of the MNIST recognition using memristive temporal kernel computing systems^20,21^ and a software-based system^1^ (single-layer FCN), showing very fast processing and the highest accuracy in this study.

Group	Accuracy	Latency in the kernel	Kernel adaptation	Network size	Image size	Etc.
This study	90% (95.1%—two layer)	$1\;\upmu s$	O	196 × 10 (196 x 38 x 10)	28 × 28	
Wei.D.Lu	85%	$10\;{{{{{\rm{m}}}}}}s$	X	88 × 10	22 × 20	14,000/2,000 Training/test set
Joshua Yang	83%	$1\;{{{{{\rm{m}}}}}}s$	X	220 × 10	22 × 20	In situ training
Software (784 × 10 FCN)	91%	-	-	784 × 10	28 × 28	

This kernel machine took 200 ns of time and ~25 pJ of energy (Supplementary Fig. S16) to process one input pulse, which is 10^3^–10^4^ times shorter and 100–400 times lower than in the previous studies^20–22^. Table 2 shows the comparison with other RC results using the diffusive memristors and the software-based single-layer FCN. This study focuses on the only memristive TK system that performs kernel adaptation and that showed the best performance in terms of accuracy and latency. Supplementary Table 4 shows the results for the case where the 2-layer FCN is used as the readout layer, and when 196 × 38 × 10 FCN is used, it offers 95.1% accuracy. The number of training parameters in this network (7828) is slightly smaller than that of the software-based FCN (7840). The readout network size of the TK system could be further decreased as the number of bits processed by the kernel (nBPK) increases, for as long as the separability for the higher nBPK is guaranteed. Supplementary Fig. S17 shows the different read currents for the 3–6 bits (8–64 input patterns). Obviously, the separability decayed as the nBPK increased, but they were still be used to recognize the MNIST dataset because not all the input patterns mattered equally. Table 3 shows the variation in the test accuracy of the MNIST dataset using the same method as above, but with different nBPKs. As the nBPK increased from 3 to 6, which was accompanied by a decrease in the required memristor number from 252 to 112, the accuracy decreased from 90.7% to 86.3% (the confusion matrices are included in Supplementary Fig. S18), which is not much lower than in the software-based FCN (784 × 10). The next section demonstrates the most crucial merit of this TK system by showing its capacity to process time-series data using medical diagnostic data.

**Table 3:** Results of the MNIST recognition while increasing the number of bits processed in the temporal kernel, showing that as nBPK increased, both the size of the used readout layer and the recognition accuracy decreased.

nBPK	Readout layer size	Accuracy
3	252 × 10	90.7%
4	196 × 10	90.1%
5	140 × 10	88.1%
6	112 × 10	86.3%

### Task optimization: medical diagnosis

Medical diagnosis often requires analyzing time-varying data and making a quick diagnosis, but there are inevitable limitations such as high dependence on operators and high variability across different medical institutions. For a more accurate and objective medical diagnosis, a universal diagnosis system adaptable to various situations is essential. Automatic medical diagnosis using deep learning has considerable potential, and several studies have been conducted on it^33–35^, but most of them rely on the conventional image classification method, such as CNN. This means that the traditional medical diagnosis system produces data images and analyzes them later, mostly ex-situ. This study suggests a method for in situ medical diagnosis in real-time using a 1M1R1C kernel. The diagnostic application consists of two sections. The first section is breast cancer diagnosis using ultrasound images, and the second section is arrhythmia diagnosis based on electrocardiogram (ECG) results. These two applications have vastly different operating signal frequencies (MHz to Hz). In this study, a system for efficient medical diagnosis was implemented by optimizing the TK system for each task.

**Fig. 4:**
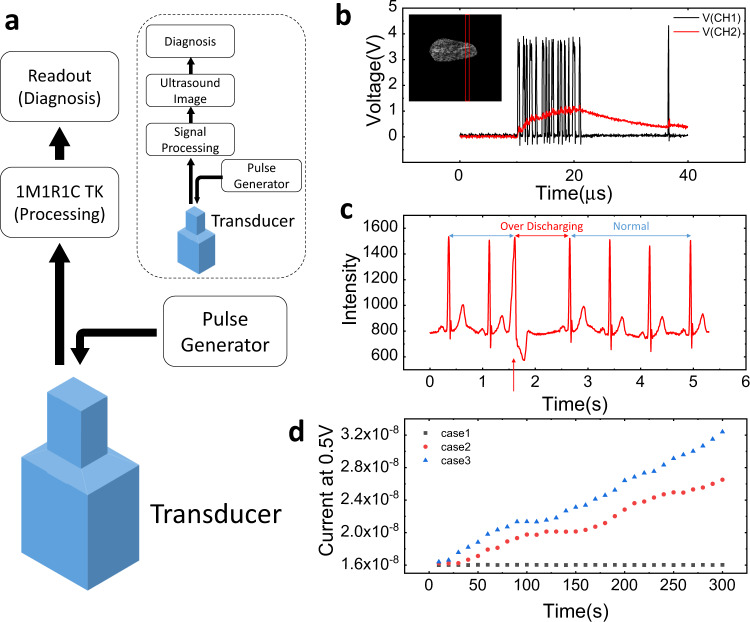
The automatic medical diagnosis system using the 1M1R1C temporal kernel and the experiment results in the two sections. **a** A system for diagnosing the malignancy of breast lesions, which is much simpler than in the existing method (inset in **a**). In this system, ultrasonic signals are applied directly to the kernel machine, so the imaging step is omitted. **b** *V–t* graph for one echo line of a benign sample (inset in Fig. 4b). **c** A part of the electrocardiogram of a patient with arrhythmia. Long intervals caused by abnormal beats discharged the capacitor, and the conductance of the memristor increased in the next pulse. **d** Five-minute temporal kernel monitoring based on the ECG of one normal patient (case 1) and two arrhythmic patients (cases 2 and 3). When arrhythmia occurred, the conductance of the memristor increased. Case 3, which had the most severe arrhythmia symptoms, showed the highest conductance.

1) Diagnosis of malignancy in breast lesions. Breast cancer is the most common cancer in women. Ultrasound is used to diagnose and monitor this disease. In contrast to the conventional CNN, where the preprocessed images are identified, the proposed TK system in this study directly uses ultrasonic raw data without an imaging process, as shown in Fig. 4a. In the conventional ultrasound diagnosis, the ultrasound is transmitted to the piezoelectric material, where electrical signals are generated. The signal processor processes these signals to generate an ultrasound image, which the operator analyzes to diagnose the disease. However, if the TK can directly process the ultrasonic signal, the imaging process can be skipped, and an automatic diagnosis will be made at the readout layer. Therefore, this system makes real-time diagnosis simpler than in the existing ultrasound diagnosis.

The dataset used in the experiment consisted of an open-access database of raw ultrasound signals acquired from malignant and benign breast lesions^36^. Each sample consisted of 510 ultrasound (10 MHz) echo lines. After they were preprocessed for measurement convenience, they were converted into pulse streams and applied to the memristor (“Methods” section). Figure 4b shows the results of the voltage-time (V-t) measurement for one echo line of a benign sample (inset in Fig. 4b, and “Methods” section). The test set consisted of 36 samples randomly extracted out of the total 100 samples, and the training set consisted of the remaining 64 samples. Readout was performed by repeating the process of randomly extracting the test set from the entire dataset 30 times, and an average accuracy of 94.6% was obtained.

This method has two main advantages over the existing ultrasound diagnosis using CNN. First, diagnosis is performed using a much simpler system without a pre-imaging process. Second, one of the major difficulties in ultrasound analysis is the presence of artifacts^33^. CNN may have difficulty in recognizing such artifacts because it performs learning and inference with the information on the artifacts. Using 1M1R1C, even with additional stimulation by artifacts, the capacitor only maintains the charging state. Therefore, the kernel state is determined by the overall contour rather than by fine artifacts, and it can show higher performance.

2) Real-time arrhythmia diagnosis. Arrhythmia is a condition in which the heart has an irregular rhythm or an abnormal heart rate. Since malignant arrhythmia can cause sudden death due to a heart attack^37^, real-time ECG monitoring and diagnosis are required. The purpose of this experiment is to implement a system capable of real-time diagnosis of arrhythmia in response to an electrical signal caused by a heartbeat. For the experiment, a part of the MIT-BIH arrhythmia database^38^ was used, and a task-optimized kernel was utilized to distinguish between arrhythmia and normal cases. A TK capable of responding to a signal with a frequency of 0.8–1.2 Hz was constructed using a 1 µF capacitor parallel to CH2. In this case, a simple temporal kernel machine composed of only one 1M1R1C kernel could be used. Figure 4c shows a part of the ECG of a patient with arrhythmia. The electrical signal is generated at approximately 0.8-s intervals, and then arrhythmia occurs at 1.6 s (marked by a red arrow). When an electrical signal from a heartbeat is applied to the kernel machine, the capacitor maintains a high charging level at a normal beat. When an arrhythmia occurs, the capacitor is discharged at a longer interval than in the normal case, and SET switching occurs in the memristor by the next pulse (Supplementary Fig. S19). Since this kernel responds only to arrhythmia, the memristor conductance can reflect the pulse of the arrhythmia patient in real-time. Figure 4d shows the results of 5-min TK monitoring based on ECG data of normal (case 1) and arrhythmic (cases 2 and 3) patients. In cases 2 and 3, 49 and 81 arrhythmias occurred, respectively. As a result, the conductance of the TK monitoring in case 3 was the highest, and the memristor conductance was clearly distinguished according to the degree of arrhythmia. This single TK system was able to detect different arrhythmia conditions in real-time with low energy using a simple 1M1R1C circuit.

## Discussion

In this study, a TK system with high kernel separability and dynamics controllability was demonstrated using a W/HfO_2_/TiN memristor. A dynamic kernel was generated by composing a 1M1R1C circuit. From asymmetric charging/discharging of the capacitor caused by the memristor, separability, which is the basic property of the TK, was achieved. In addition, the manner in which the kernel reacted to the input signal was modified by changing various parameters such as the load resistor, capacitance, pulse width, and pulse height. Using these characteristics, the TK system was optimized to perform static data-based MNIST recognition applications and sequential data-based medical diagnoses (ultrasound diagnosis and ECG-based diagnosis). For the MNIST recognition, a task-optimized system was used to improve the separability of the inputs that frequently appeared in the dataset. Furthermore, the tradeoff between the reduction of the readout layer size and the performance was confirmed by increasing the nBPK. TK system-aided diagnosis was conducted for two situations with contrasting input frequencies (1 Hz and 10 MHz). By implementing a kernel configuration suitable for each task (kernel adaptation), the excellent performance was achieved. In particular, the most crucial point of this study is its demonstration that dynamic signals with vastly different time constants can be well distinguished by changing the resistor or capacitor added to the circuit using only one type of memristor.

The two types of hardware needed to implement the 1M1R1C TK system and analysis on the area/cell are shown in Supplementary Fig. S20. In both cases, using a metal-insulator-semiconductor capacitor, the capacitance can be adjusted by modifying the R and pulse height (Supplementary Fig. S21). Therefore, it is expected that the fabrication of the hardware for the array configuration will be simple and that the TK dynamics can easily be changed even in the fabricated hardware.

## Methods

### Memristor fabrication

The array of cross-bar-type W/HfO_2_/TiN memristors was fabricated. A 50 nm-thick TiN layer was sputtered (Endura, Applied Materials) on an SiO_2_/Si substrate, and the TiN layer was patterned into a line shape to form a BE. The 2–10 µm wide TiN BEs were patterned using conventional photolithography and the dry-etching system. After the patterning, the residual photoresist was removed with acetone and cleaned sequentially with deionized water. Then 4 nm HfO_2_ was deposited using atomic layer deposition (ALD) at a 280 °C substrate temperature using a traveling-wave-type ALD reactor (CN-1 Co. Plus 200). A tetrakis-ethlylmethylamido hafnium (TEMA-Hf) and O_3_ were used as precursors for Hf and oxygen, respectively. On the HfO_2_ layer, 50-nm-thick W TEs were sputtered using the MHS-1500 sputtering system and patterned into 2–10 µm wide lines using the conventional lift-off process. After the fabrication, the WHT device was analyzed using x-ray photoelectron spectroscopy (XPS, AXIS SUPRA, Kratos) and energy-dispersive x-ray spectroscopy (EDS, JEOL, JEM-ARM200F) to observe the formation of the tungsten oxide layer. Cross-sectional transmission electron microscope (TEM) images of the WHT memristor were observed using scaning transmission electron microscopy (STEM, JEOL, JEM-ARM200F).

### Modified National Institute of Standards and Technology database

The dataset, the Modified National Institute of Standards and Technology (MNIST) database^39^, is a large database of handwritten digit images. It is commonly used for training and testing of image processing systems such as artificial neural networks. The database was created by “remixing” the digit samples from NIST’s original datasets^40^. This database consists of 60,000 training samples and 10,000 test samples.

### Experimental setup for the 1M1R1C TK computing

To compose the temporal kernel circuit, the WHT device with an area of 10 µm × 10 µm was connected to the pulse generator (PG, Agilent 81110 A) and an oscilloscope (OSC). A 1M1R1C circuit was constructed by adding a load resistor to the circuit and setting the resistance values of CH1 and CH2 in the OSC to 50 Ω and 1 MΩ, respectively. A semiconductor parameter analyzer (SPA, Hewlett-Packard 4145B) was connected to the WHT device to monitor the DC sweeps. To process the static and sequential data, the device states after the pulse streams were measured. After the measurement, the device was reset to the HRS state and the process was repeated. The TK state was constructed based on the recorded device states, and the readout layer was trained based on it.

### PyTorch simulation for the readout layer of the TK system and 784 × 10 FCN

The logistic regression algorithm was used to train the readout layer for the MNIST recognition and breast lesion classification. The TK state (**x**) in the form of an *n* × 1 vector (*n* = 784–112 for the MNIST recognition and *n* = 510 for the breast lesion classification) was multiplied by the weight matrix (**W**) of the readout layer to yield the weighted sum (**z**).

1 $${{{{{\bf{z}}}}}}={{{{{{\bf{W}}}}}}}^{{{{{{\rm{T}}}}}}}{{{{{\boldsymbol{\bullet }}}}}}{{{{{\bf{x}}}}}}$$

The weighted sum was applied to the following softmax function to yield an output ($\hat{{{{{{\bf{y}}}}}}}$):

2 $${\hat{{{{{{\bf{y}}}}}}}}_{j}={{{{{\rm{\sigma }}}}}}{\left({{{{{\bf{z}}}}}}\right)}_{j}=\frac{{{{{{{\boldsymbol{e}}}}}}}^{{{{{{{\boldsymbol{z}}}}}}}_{{{{{{\boldsymbol{j}}}}}}}}}{{\sum }_{{{{{{\boldsymbol{k}}}}}}={{{{{\bf{1}}}}}}}^{{{{{{\boldsymbol{n}}}}}}}{{{{{{\boldsymbol{e}}}}}}}^{{{{{{{\boldsymbol{z}}}}}}}_{{{{{{\boldsymbol{k}}}}}}}}}\;{for}\;{j}=1,\ldots ,n.$$

The sum of the elements of the output vector became 1 and the output of the softmax function was perceived as a ‘probability.’ The cross-entropy loss was used for the loss function, which is defined as

3 $${{{{{{\rm{loss}}}}}}}=-\frac{1}{N}\mathop{\sum }\limits_{i=1}^{N}\left[{{{{{{\boldsymbol{y}}}}}}}_{i}{\log }\left({\hat{{{{{{\bf{y}}}}}}}}_{i}\right)+\left(1-{{{{{{\boldsymbol{y}}}}}}}_{i}\right){\log }\left(1-{\hat{{{{{{\bf{y}}}}}}}}_{i}\right)\right],$$

wherein N is the number of samples, and ${{{{{{\boldsymbol{y}}}}}}}_{i}$ is the target output for input ${{{{{{\boldsymbol{x}}}}}}}_{i}$. To minimize the loss, a gradient-descent-based Adam optimizer^41^ was identically used for the readout layer and 784 × 10 FCN. Full-batch-type learning of the readout layer and 784 × 10 FCN was performed in PyTorch.

### TK system for medical diagnosis

(1) Ultrasound-based breast lesions diagnosis: Each sample in the database consisted of 510 ultrasound (10 MHz) echo lines, and the length of each echo line was different for each sample (100–300 µs). For measurement convenience, samples in which breast lesions appeared within 40 μs were used for learning and inference. The raw ultrasound data were binarized and converted into 100 ns pulses, which corresponded to a 10 MHz frequency. Pulse streams that consisted of 400 100 ns long pulses were applied to the memristor. The measurement setup was set at a 3.5 V pulse height, 4 V reference pulse height, and a 30 kΩ load resistance. The use of relatively large load resistance and a short pulse length made the kernel sensitive to consecutive pulses, and the effect of the signal pulses remained until the reference pulse (Fig. 4b). After the measurement, the 510 × 2 readout layer was trained based on the TK state that consisted of the 510 kernel responses for each input. (2) ECG-based arrhythmia identification: The measurement was performed under the conditions of no *R*_L_, a 2.5 V pulse height, a 200 ms length, and a 1 µF capacitor.

## Supplementary information

Supplementary Information

## Data Availability

All the relevant data are available from the corresponding authors upon reasonable request.

## Code availability

Computational results were obtained by using Pytorch software programs. Pytorch was used to perform the calculation of the readout layer. All the relevant codes are available from the corresponding authors upon reasonable request.
