# Supplementary material for: Time-varying data processing with nonvolatile memristor-based temporal kernel
Source: Nat Commun. 2021 Sep 30;12:5727. doi: 10.1038/s41467-021-25925-5 (PMC8484437; doi:10.1038/s41467-021-25925-5)
Supplement: Supplementary file 1 — Supplementary Information [file 41467_2021_25925_MOESM1_ESM.pdf]

SUPPLEMENTARY INFORMATION

Time-varying data processing with nonvolatile memristor-based temporal kernel

Yoon Ho Jang[1,2], Woohyun Kim[1,2], Jihun Kim[1,2], Kyung Seok Woo[1,2], Hyun Jae Lee[1,2], Jeong Woo Jeon[1,2], Sung Keun Shim[1,2], Janguk Han[1,2], and Cheol Seong Hwang[1,2†]*

[1]Department of Materials Science and Engineering College of Engineering, Seoul National University, Seoul, 08826, Republic of Korea
[2]Inter-university Semiconductor Research Center, Seoul National University, Seoul, 08826, Republic of Korea

[†] Corresponding author (e-mail: cheolsh@snu.ac.kr)

Supplementary Figures

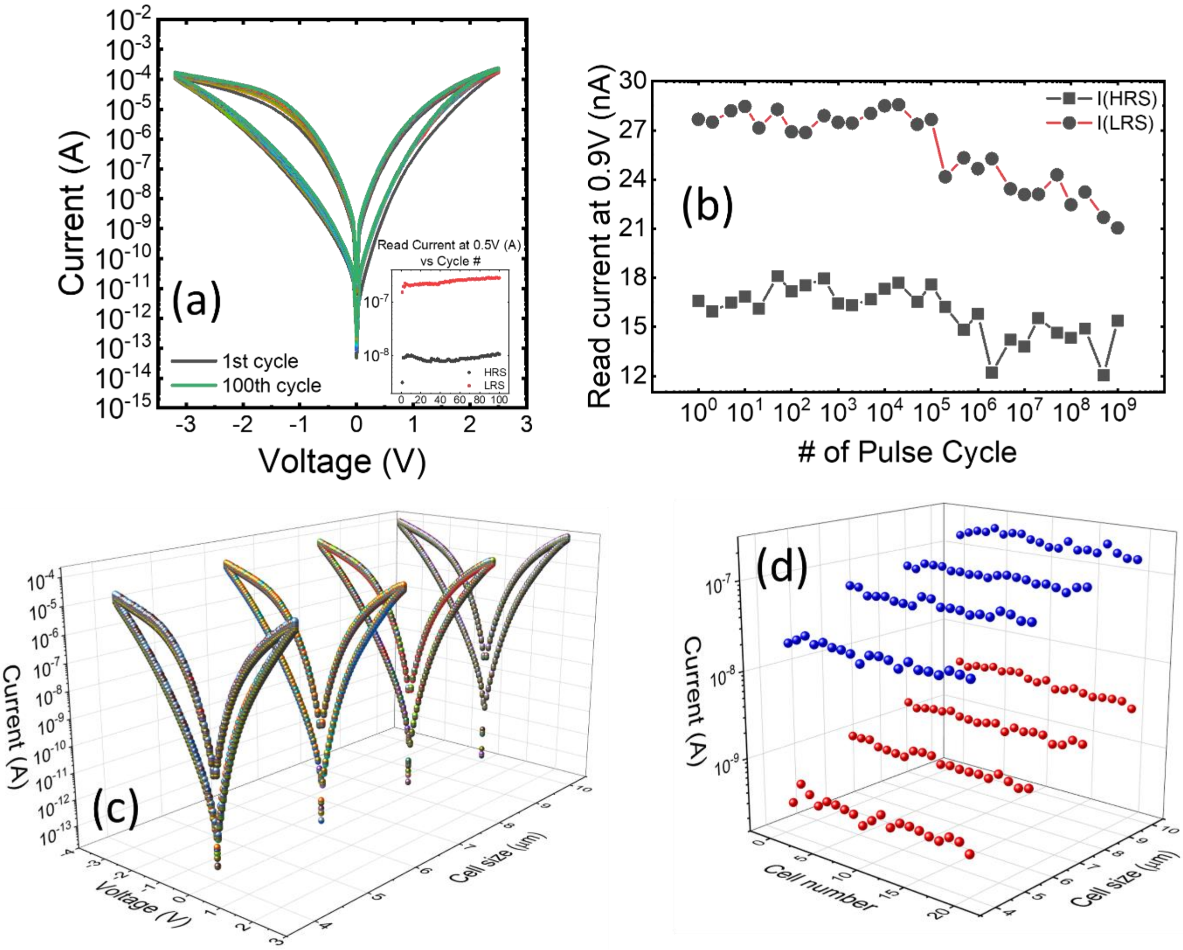

Supplementary Figure S1: Experimental results on device reliability and reproducibility. **a)** Cycle to cycle variation of the WHT memristor. Except for the first cycle out of 100 DC cycles (2.5 ~ -3.2 V), there was a slight variation in the I-V curve. The inset of (**a**) shows the read current at 0.5V for each cycle number. **b)** Endurance of the WHT memristor. The WHT memristor showed stable resistive switching behavior during ~10^5 pulse cycles. For endurance measurement, a 3.3 V height 1 µs width SET pulse and -3.35V height 1.5 µs width RESET pulse were used. The read current was recorded with DC read at 0.9 V and a WHT memristor with 4 µm cell size was used for measurement. **c, d)** Cell to cell variation of the WHT memristor. A total of 80 devices were measured with 20 devices each of 4 µm x 4 µm, 6 µm x 6 µm, 8 µm x 8 µm, and 10 µm x 10 µm. An I-V curve was obtained in each device through a 2.5 V ~ -3.2

V DC cycle (**c**), and the read current was extracted at 0.5 V of each I-V curve (**d**). Data shown in red is read current in HRS and data shown in blue is read current in LRS.

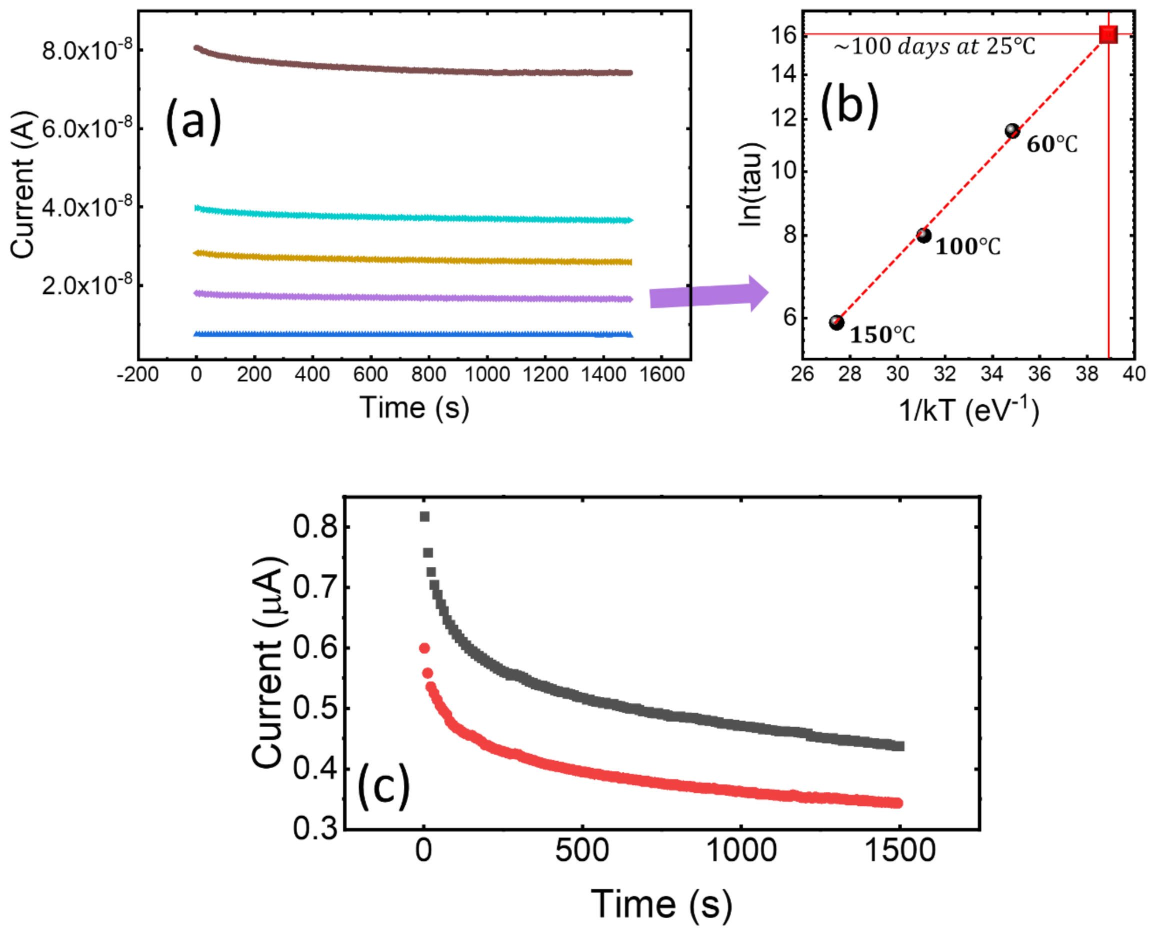

Supplementary Figure S2: Retention measurement result of the WHT device. The WHT device has a nonvolatile characteristic in the low conductance range (**a**) and a retention time of about 100 days at 25 ℃, which is the result of extrapolation based on 60 ~ 150 ℃ retention data (**b**). Meanwhile, the WHT device has a volatile characteristic in a high conductance region (**c**). This is because the trap depth exerted on the electrons is different according to the conductance state (trapped electron density). In the above case, the trapped electron density was increased by increasing pulse height. Then, the relatively easier detrapping of the heavily trapped WHT device induced the decay of conductance with time. This can be used as the fading memory.

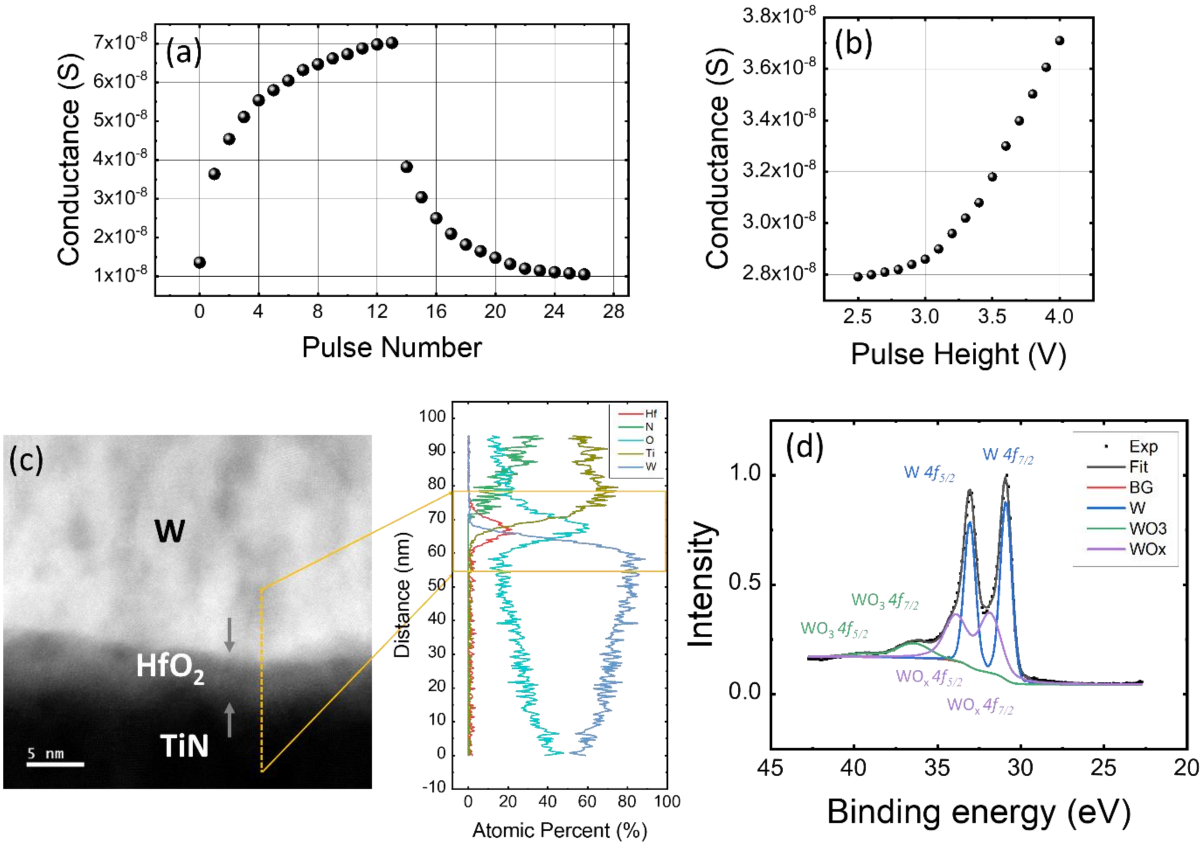

Supplementary Figure S3: Analysis of the AC characteristics, and device structure of the W/HfO_2/TiN memristor. **a)** Changes in conductance of memristor according to pulse number. Pulse number 1~13 correspond to SET pulse, 14~26 correspond to RESET pulse, and read voltage was 0.5 V. The SET and RESET pulse heights were 4 V and -4 V, respectively, and the width of both was 200 μs. **b)** The conductance of the memristor according to the 2.5~4 V SET pulse height. Multilevel switching is possible for both SET and RESET, but the change in conductance according to the pulse number is non-linear (**a**). Also, the change in conductance according to the pulse height is non-linear as the pulse height decreases (**b**). Both nonlinearities were used for the non-linear transformation of the input in the temporal kernel. **c)** Scanning transmission electron microscopy (STEM) cross-sectional image and energy-dispersive x-ray spectroscopy (EDS) analysis results (right portion) of the fabricated W/HfO_2/TiN memristor with a depth profile. **d)** XPS spectra of the W 4f region with a depth profile and fitting results for the W/HfO_2/TiN memristor. The square dot shows the measurement result (Exp), and the

black and red lines show the fitting result (Fit) and back ground (BG), respectively. Blue, green, and purple lines show XPS peaks of tungsten (W), tungsten oxide (WO_3), and tungsten suboxide (WO_x), respectively. The sample was measured immediately after the deposition. **c, d** show that tungsten oxide was generated in the memristor.

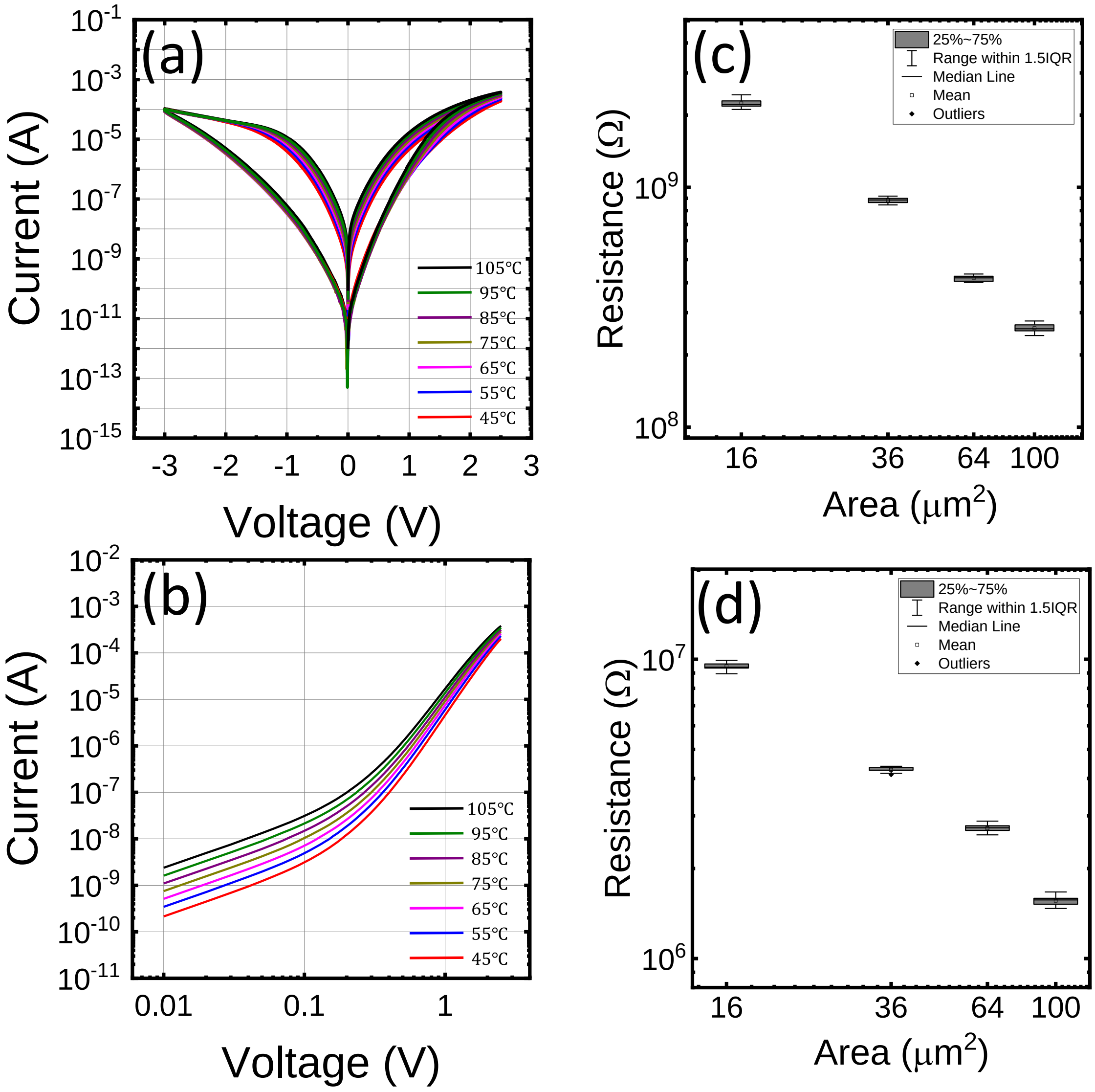

Supplementary Figure S4: The effects of the temperature and the cell area on the electrical properties of the device. **a)** The I-V curve at various temperatures (45~105 ℃). **b)** The I-V graph of the LRS at various temperatures (45~105 ℃). **c, d)** The cell area dependence of the resistance measured in 10 devices in HRS (**c**) and LRS (**d**).

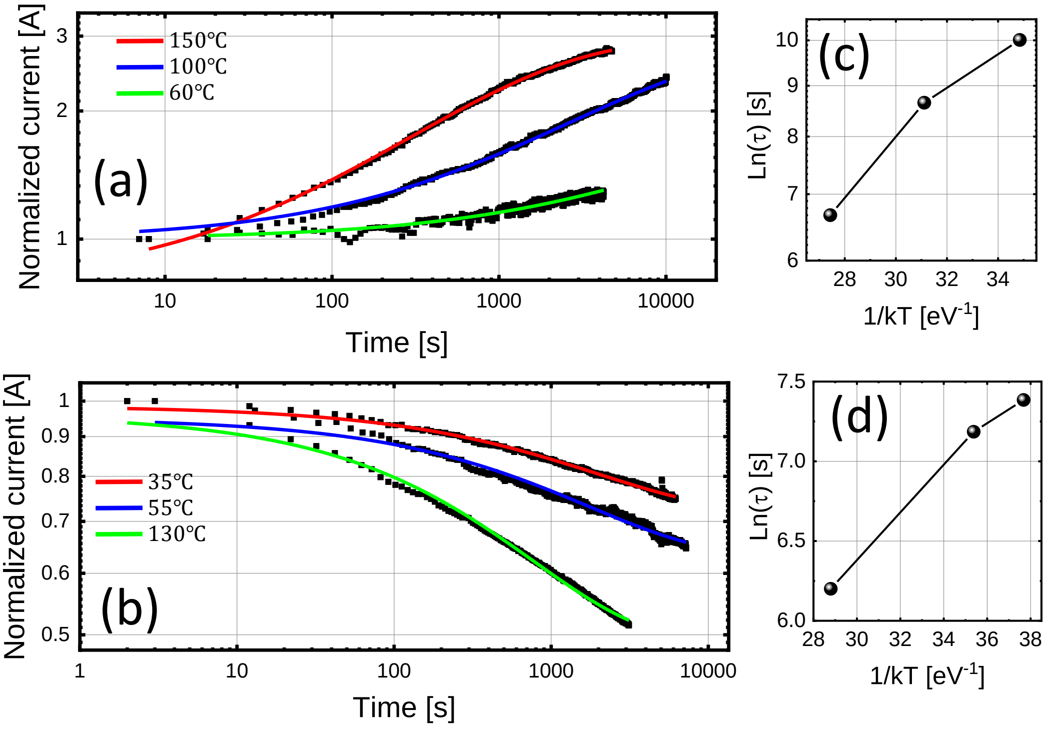

Supplementary Figure S5: The trap depth of the WHT memristor calculated from the time-dependent current-relaxation characteristics of the on and off states at various temperatures. For this test, the current was measured at the 0.5 V read voltage and the temperature was varied from 35 ℃ to 150 ℃. **a, b)** The relaxation curves at various temperatures of the HRS (**a**) and LRS (**b**). Here, the read current was normalized to the initial current at t = 0. The data show that the read current rose (**a**) and decayed (**b**) over time as the trapped electrons were being trapped (**a**) and detrapped (**b**). These relaxation curves were fitted into the stretched exponential function $[f_\beta(t) = Ae^{-\left(\frac{t}{\tau}\right)^\beta} + B]$ to attain the time constant (τ) at each temperature. **c, d)** The Arrhenius plots of ln (τ) versus 1/kT of the HRS and LRS cases. The analysis showed 0.45 eV and 0.13 eV activation energy, which correspond to the trap depth for the HRS and LRS, respectively, of the system.

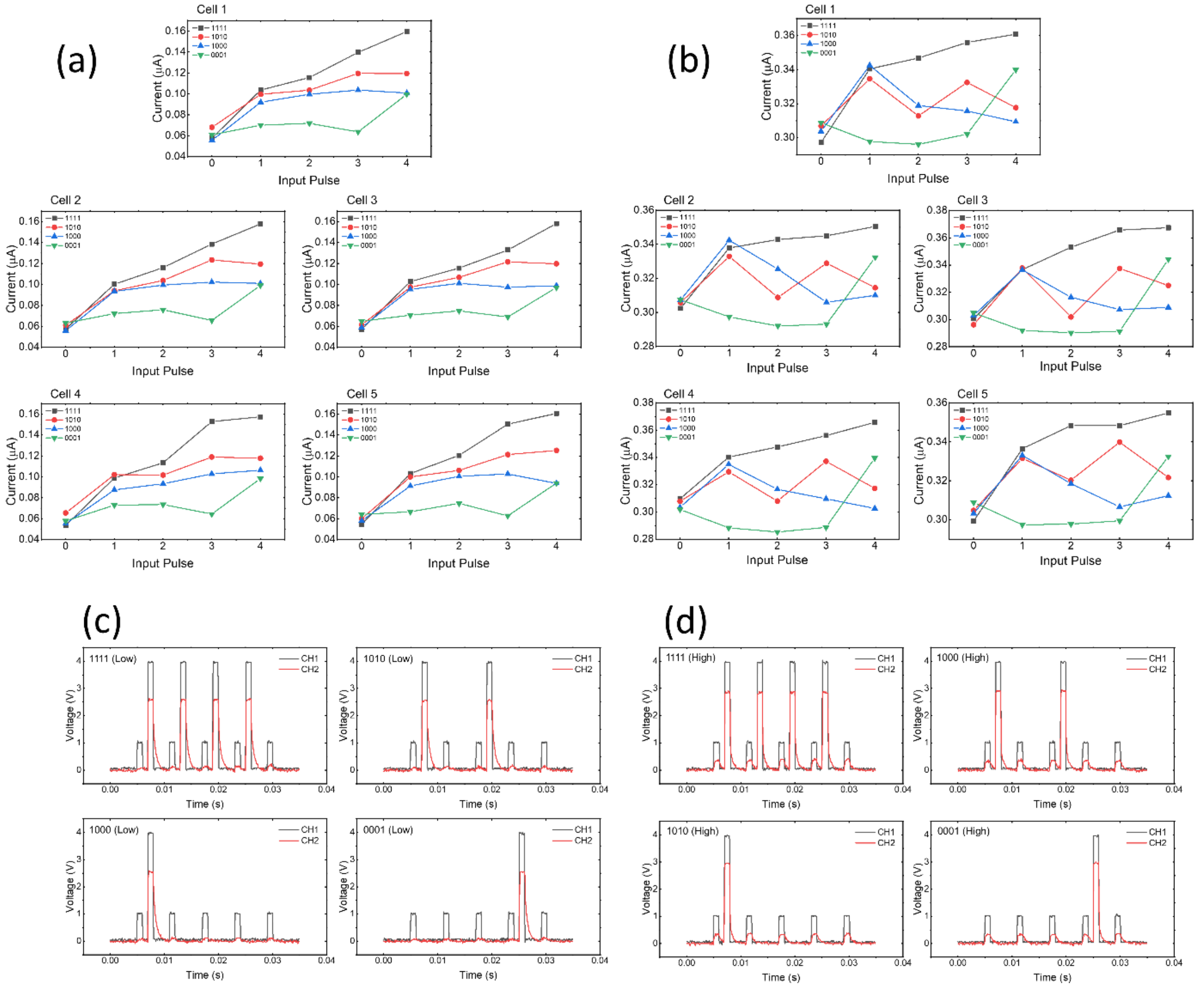

Supplementary Figure S6: Fading memory test of the WHT memristor at the low and high conductance level. **a**) Response of the 1M1R1C kernel machine to input patterns of '1111', '1010', '1000', and '0001' in the low conductance range. In the low conductance region, the WHT memristor has nonvolatile characteristics, so the effect of the high signal is accumulated and the fading memory is not implemented. In contrast, the WHT memristor has a volatile characteristic in a high conductance region, and a fading memory is implemented in this region (**b**). **c, d**) Voltage applied to CH1 and CH2 for the input patterns of '1111', '1010', '1000', and '0001' in the low (**c**) and high (**d**) conductance level of the cell 1. During the measurement, 180 pF capacitor and 390 Ω resistor were used for the 1M1R1C kernel machine. 4 V height 1 µs width pulse was used as signal pulse and 1 V height 1 µs width pulse was used as the read pulse.

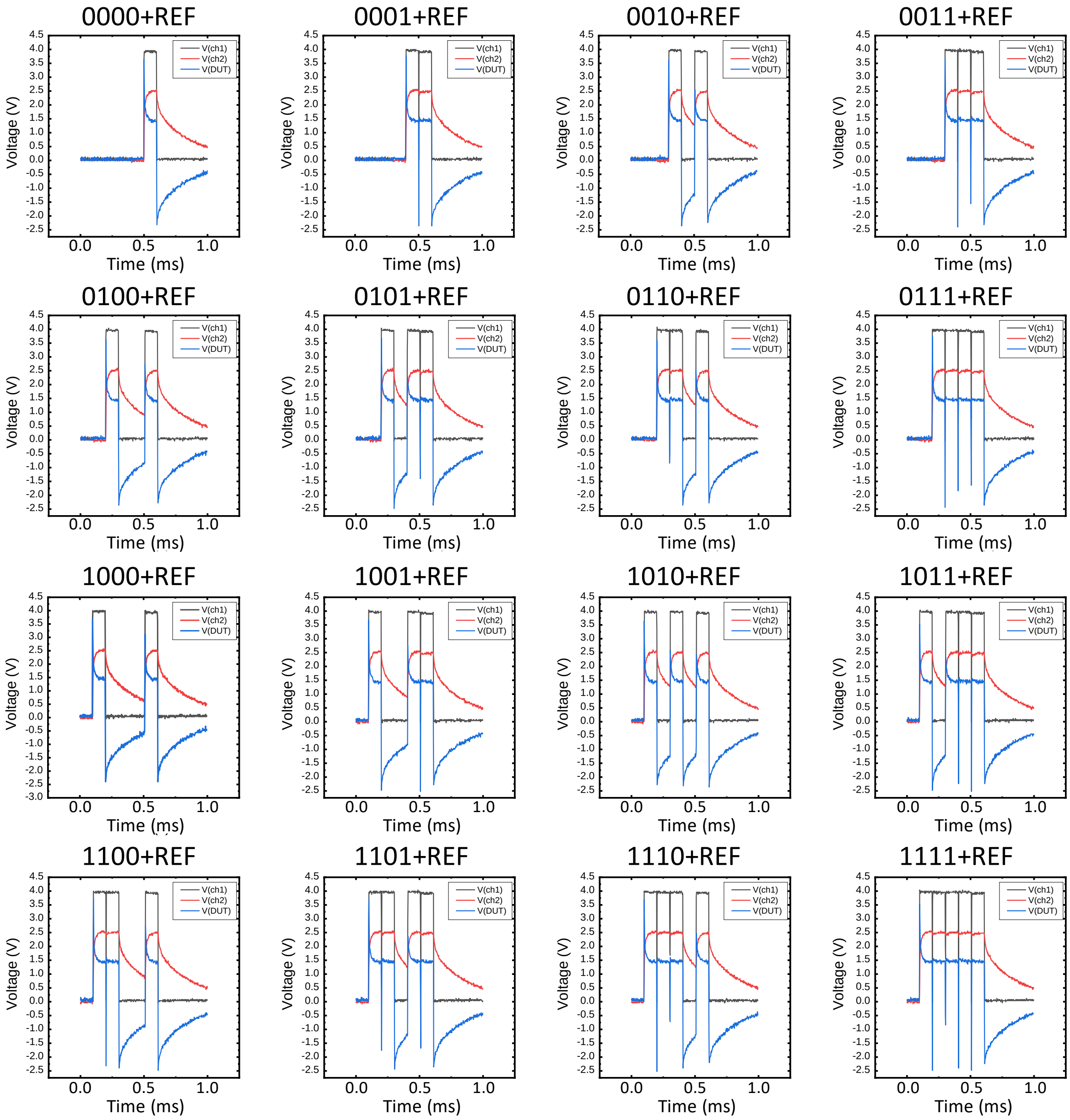

Supplementary Figure S7: The V-t graphs for the '0000'~'1111' inputs under the conditions of a 1 MΩ R_L with a 4 V signal pulse height and a 100 µs width, and a 4 V REF pulse height and a 100 µs REF pulse width.

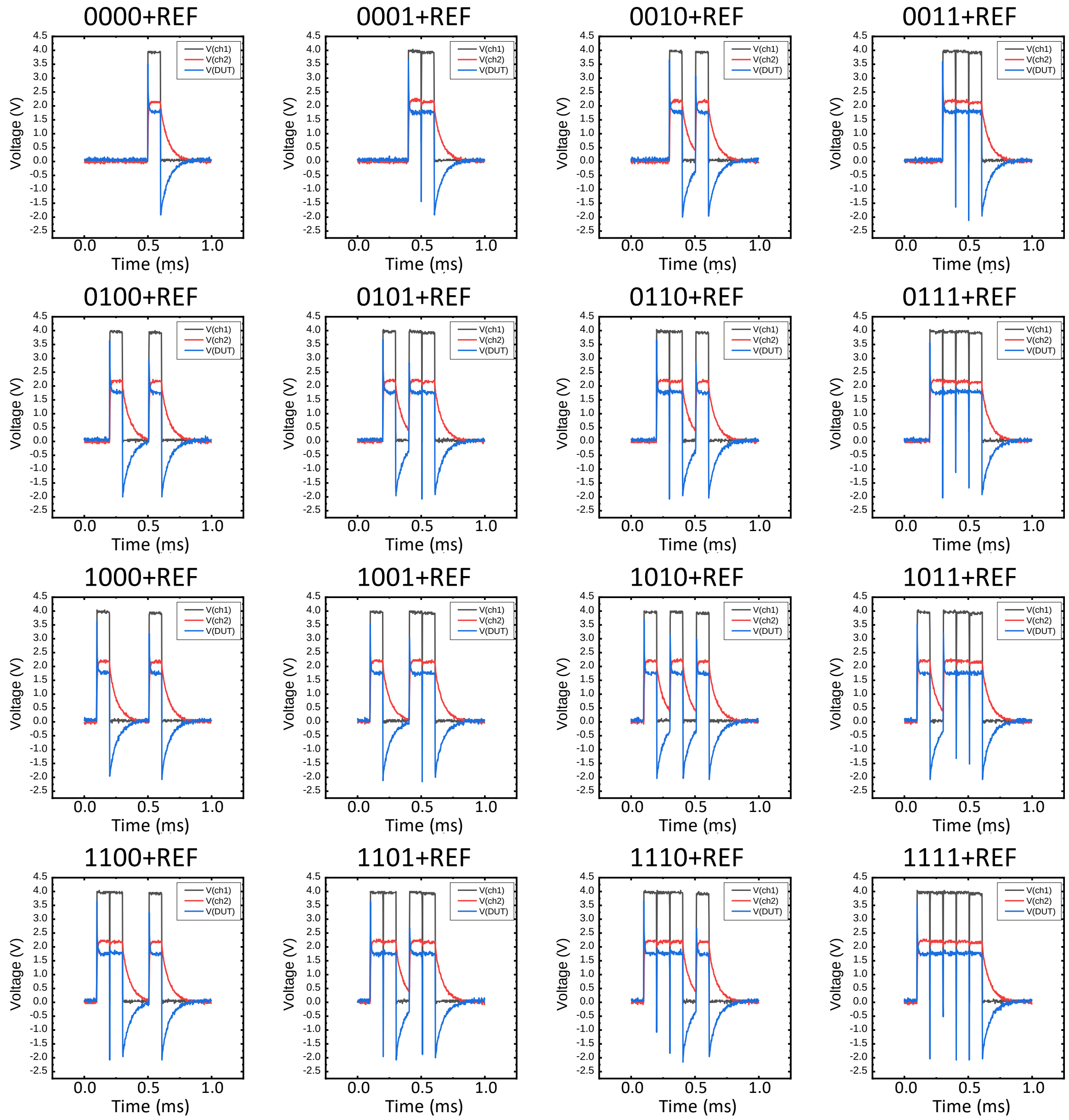

Supplementary Figure S8: The V-t graphs for the '0000'~'1111' inputs under the conditions of a 120 kΩ R_L with a 4 V signal pulse height and a 100 µs width, and a 4 V REF pulse height with a 100 µs width.

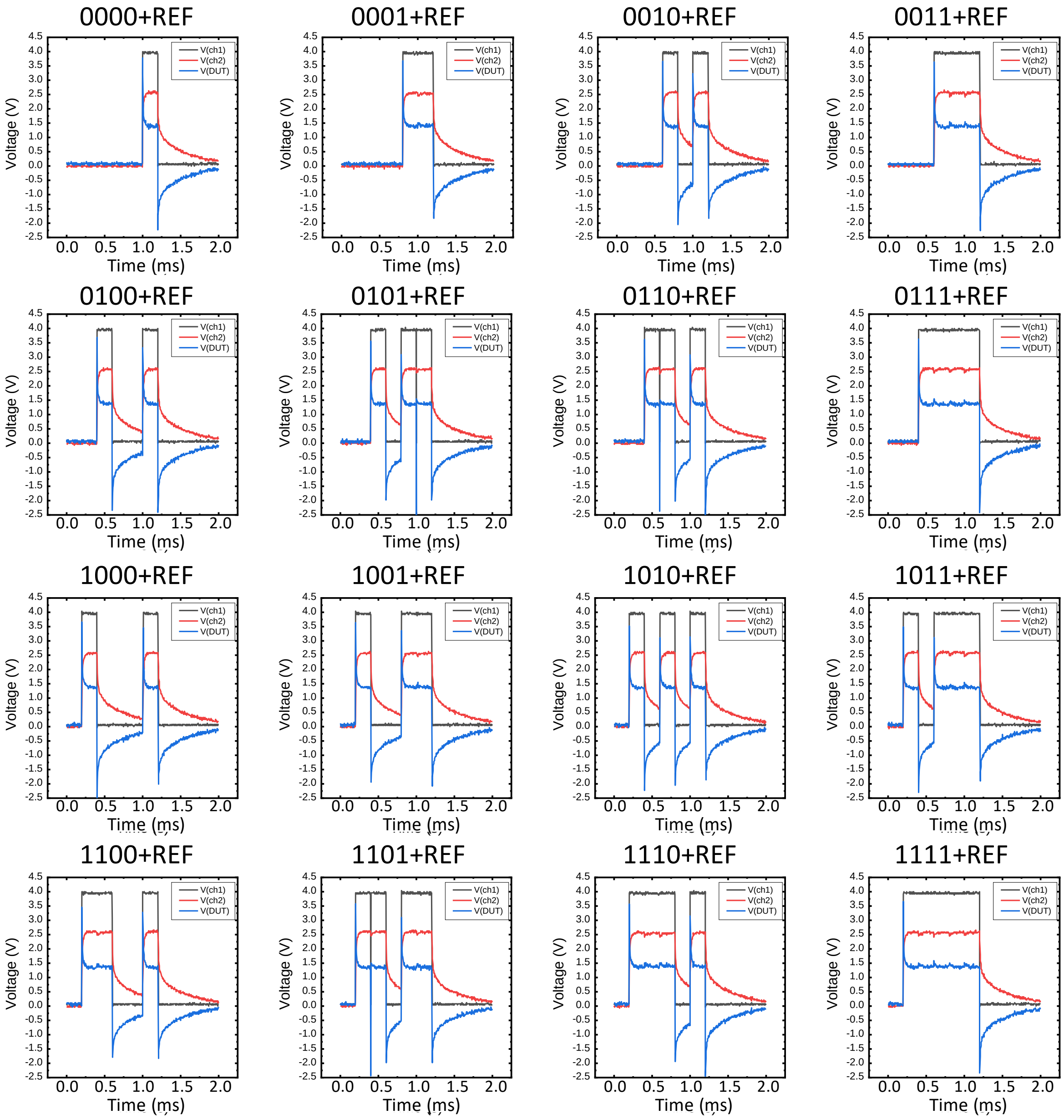

Supplementary Figure S9: The V-t graphs for the '0000'~'1111' inputs under the conditions of 1 MΩ R_L with a 4 V signal pulse height and a 200 µs width, and a 4 V REF pulse height with a 200 µs width.

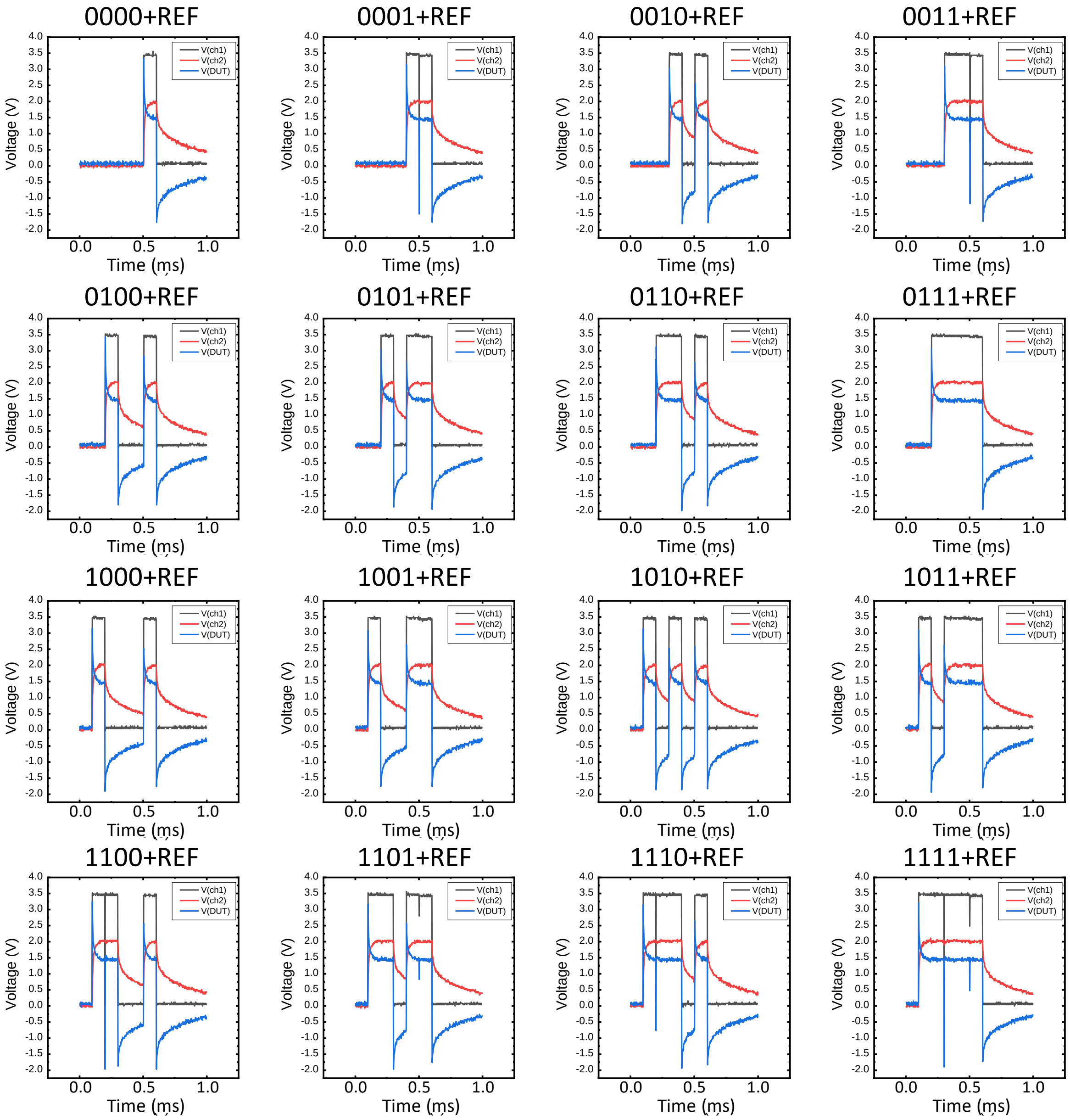

Supplementary Figure S10: The V-t graphs for the '0000'~'1111' inputs under the conditions of a 1 MΩ R_L with a 3.5 V signal pulse height and a 100 µs width, and a 3.5 V REF pulse height and a 100 µs width.

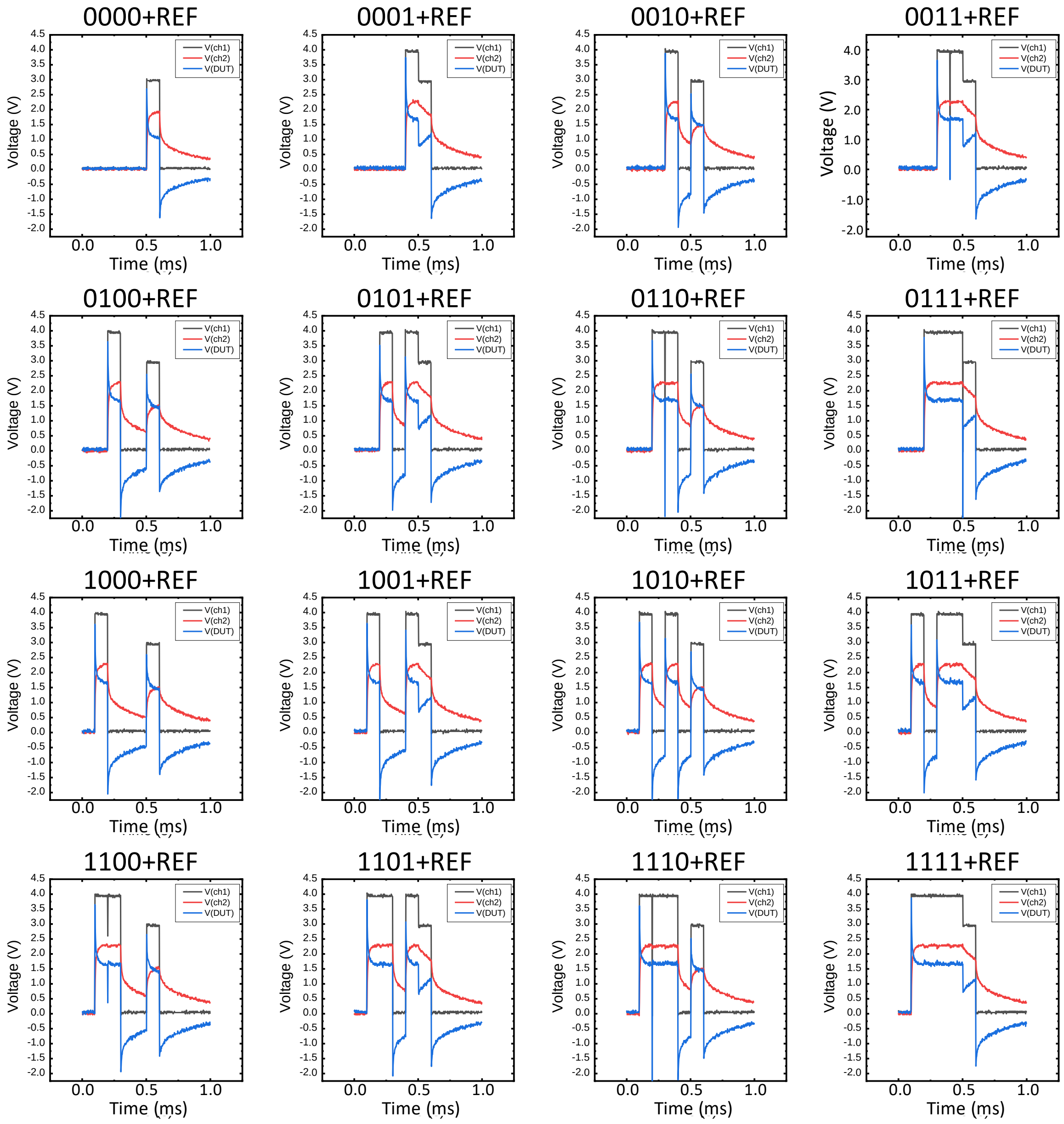

Supplementary Figure S11: The V-t graphs for the '0000'~'1111' inputs under the conditions of a 1 MΩ R_L with a 4 V signal pulse height and a 100 µs width, and a 3 V REF pulse height and a 100 µs width.

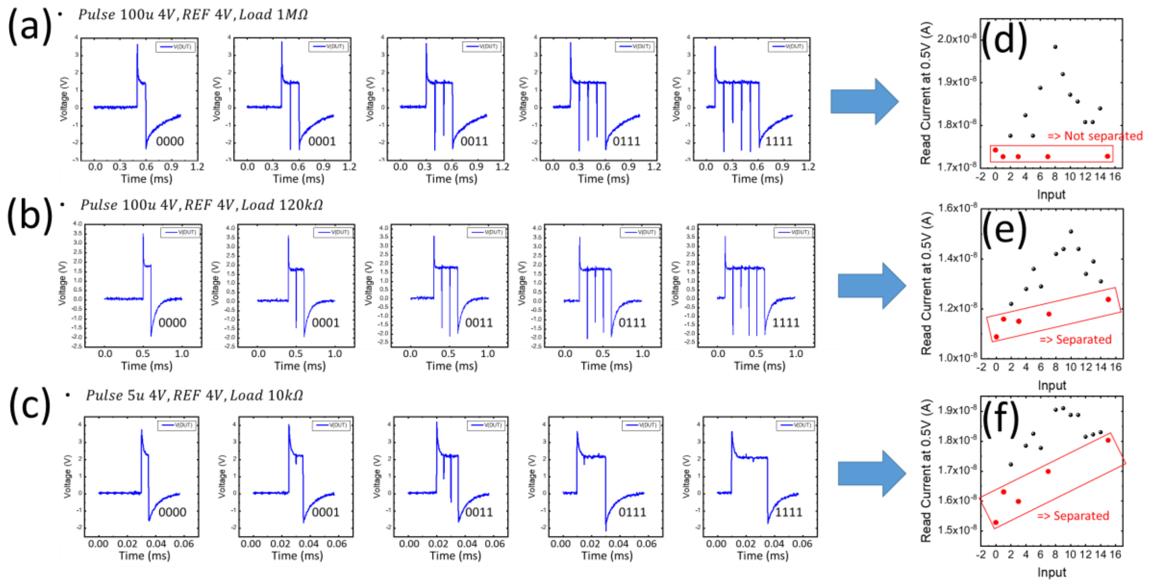

Supplementary Figure S12: Analysis of the separation of inputs that generated net 1 spikes ('0000', '0001', '0011', '0111', and '1111'). From the conditions of a 4 V signal pulse height and a 100 μs width, and a 4 V REF pulse height and a 100 μs width, R_L varies from 1 MΩ to 10 kΩ. **a-c)** The V-t graphs for the inputs that generated net 1 spikes when 1 MΩ, 120 kΩ, and 10 kΩ R_L, were used. **d-f)** The read current of the memristor for the '0000~1111' inputs under the conditions in **a-c**. When the 1 MΩ R_L was used, since the voltage distributed to the memristor was small, SET switching did not occur after the first spike (**a**). Therefore, the responses to the inputs that generated net one spike (0, 1, 3, 7, and 15) were not separated (**d**). As R_L decreased, the voltage distributed to the memristor increased (**b-c**), and thus, the responses to the corresponding inputs were separated (**e and f**).

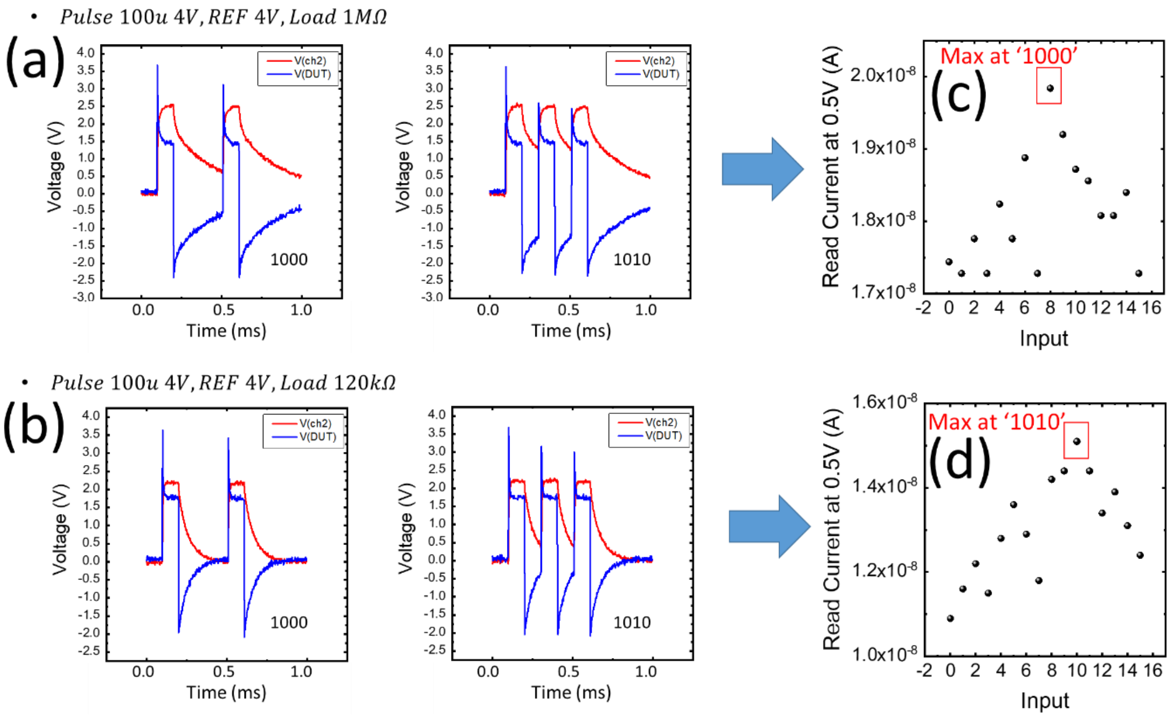

Supplementary Figure S13: Analysis of the input that caused maximum conductance. **a-b)** The V-t graphs for the '1000' and '1010' inputs under the conditions of a 4 V signal pulse height and a 100 µs width, and a 4 V REF pulse height and a 100 µs width 100, and a 1 MΩ R_L for **a** and 120 kΩ for **b**. **c-d)** The read current of the memristor for the '0000~1111' inputs under the conditions in **a-b**. Since the large R_L caused slow discharging, a sufficient interval after the first spike is necessary to generate a spike that can cause large SET switching. Under the conditions in **a**, maximum conductance occurred at the '1000' input due to the slow discharging by the 1MΩ R_L (**c**). On the other hand, under the conditions in **b**, second and third spikes of sufficient magnitude to cause SET switching occurred at the '1010' input due to the fast discharging by the 120 kΩ R_L. Therefore, maximum conductance occurred at the '1010' input (**d**).

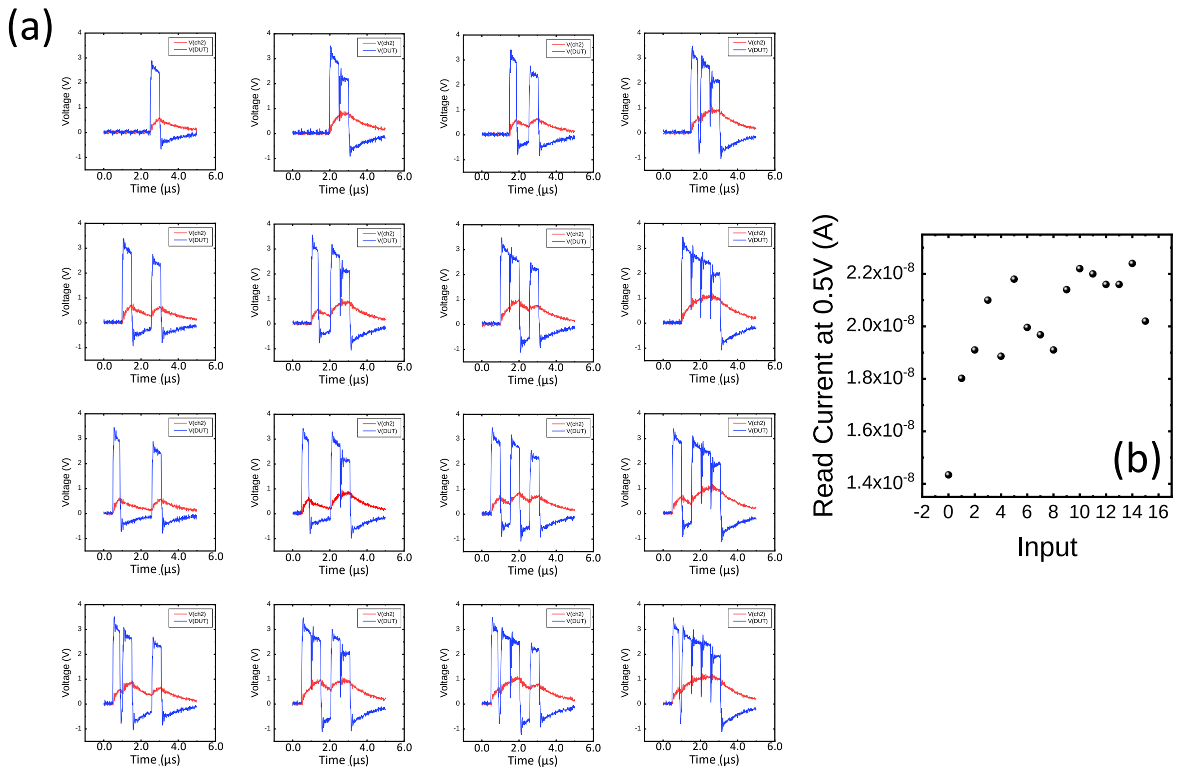

Supplementary Figure S14: a) The V-t graphs for the '0000'~'1111' inputs under the conditions of 10 kΩ R_L with a 3.5 V signal pulse height and a 500 ns width, and a 3 V REF pulse height and a 500 ns width. **b)** The read current of the memristor for the '0000'~'1111' inputs. Insufficient charging further increased the separability for the consecutive high signals since the capacitor was not fully charged even though consecutive high signals were applied. This is suitable for situations in which consecutive signals mainly appear, such as in MNIST.

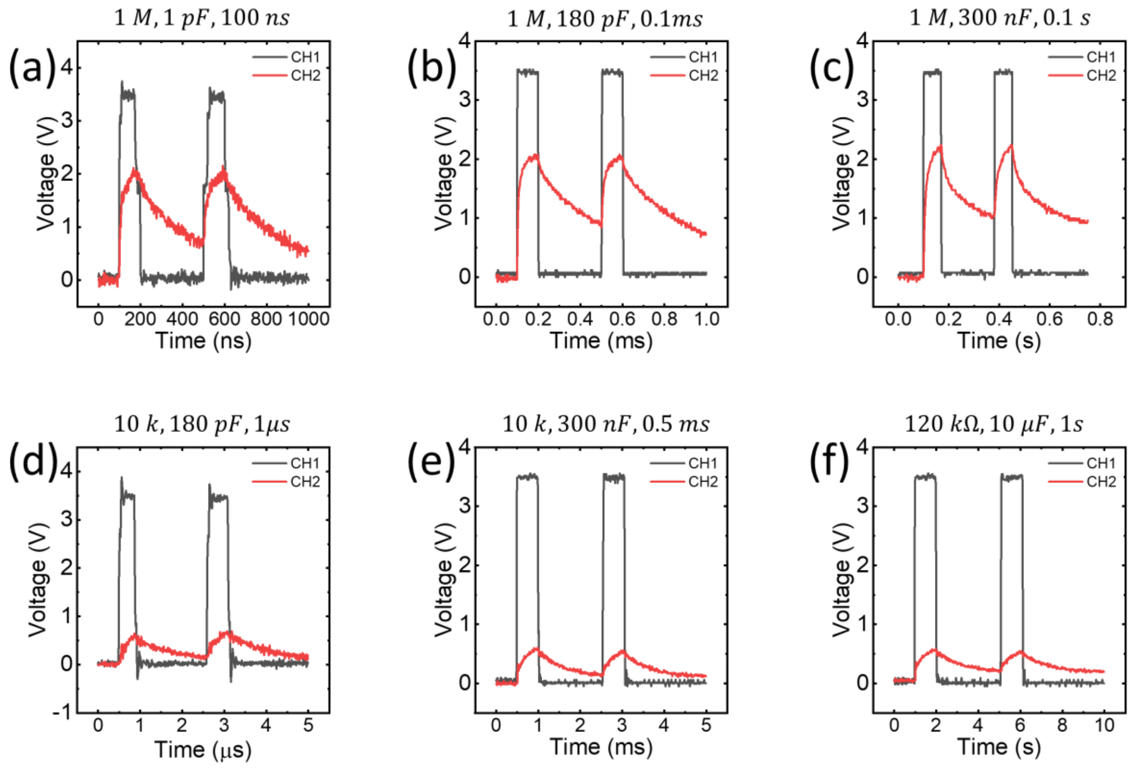

Supplementary Figure S15: Temporal kernels with different time constants (100 ns ~ 1 s). Load resistance, parallel capacitance, and input interval used in each temporal kernel are indicated in each figure. **a-e)** The V-t graphs for the '1000' input under the condition of a 3.5 V signal pulse height. **a-c** represents a temporal kernel with similar characteristics to the temporal kernel in Fig. 3a of main text, but with a different time constant. **d-f** represents a temporal kernel with similar characteristics to the temporal kernel in Fig. 3f of main text, but with a different time constant.

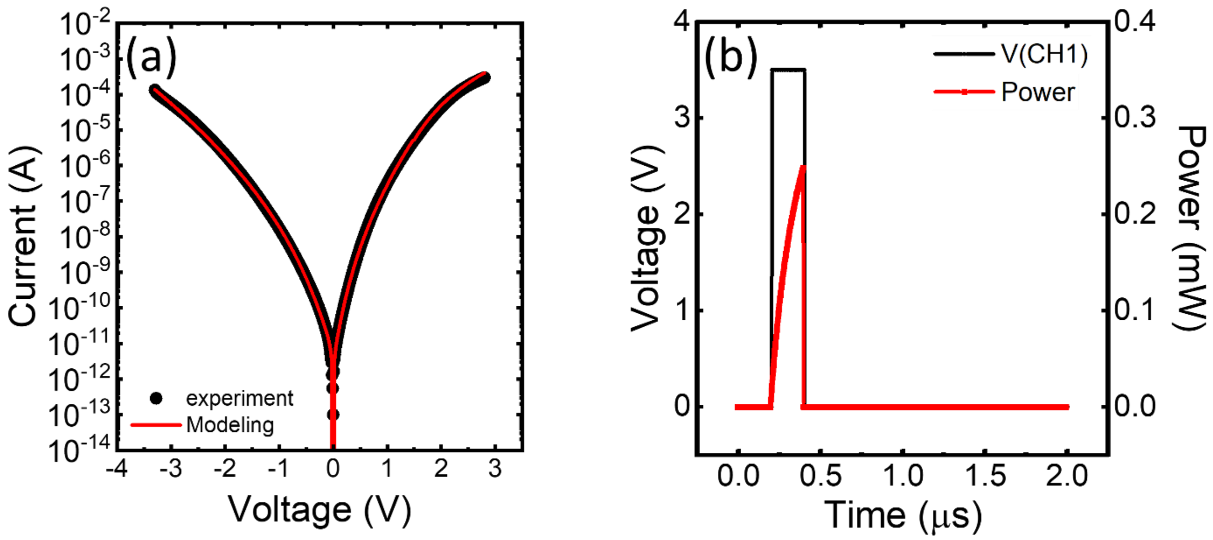

Supplementary Figure S16: Result of the I-V curve fitting for the WHT memristor and power consumption in the 1M1R1C kernel machine during processing one input. **a**) I-V curve fitting of the WHT memristor (HRS state) based on the conduction mechanisms. **b**) Power consumption in the 1M1R1C kernel machine (Fig. 3f kernel condition) during input processing. Since the resistance of the WHT memristor is dependent on the voltage, the current passing through the memristor was obtained with the HSPICE simulation using the result of the I-V curve fitting in **a**. The energy ($\int_t \ Power(t) \cdot dt$) consumed to process one input was calculated as ~25 pJ.

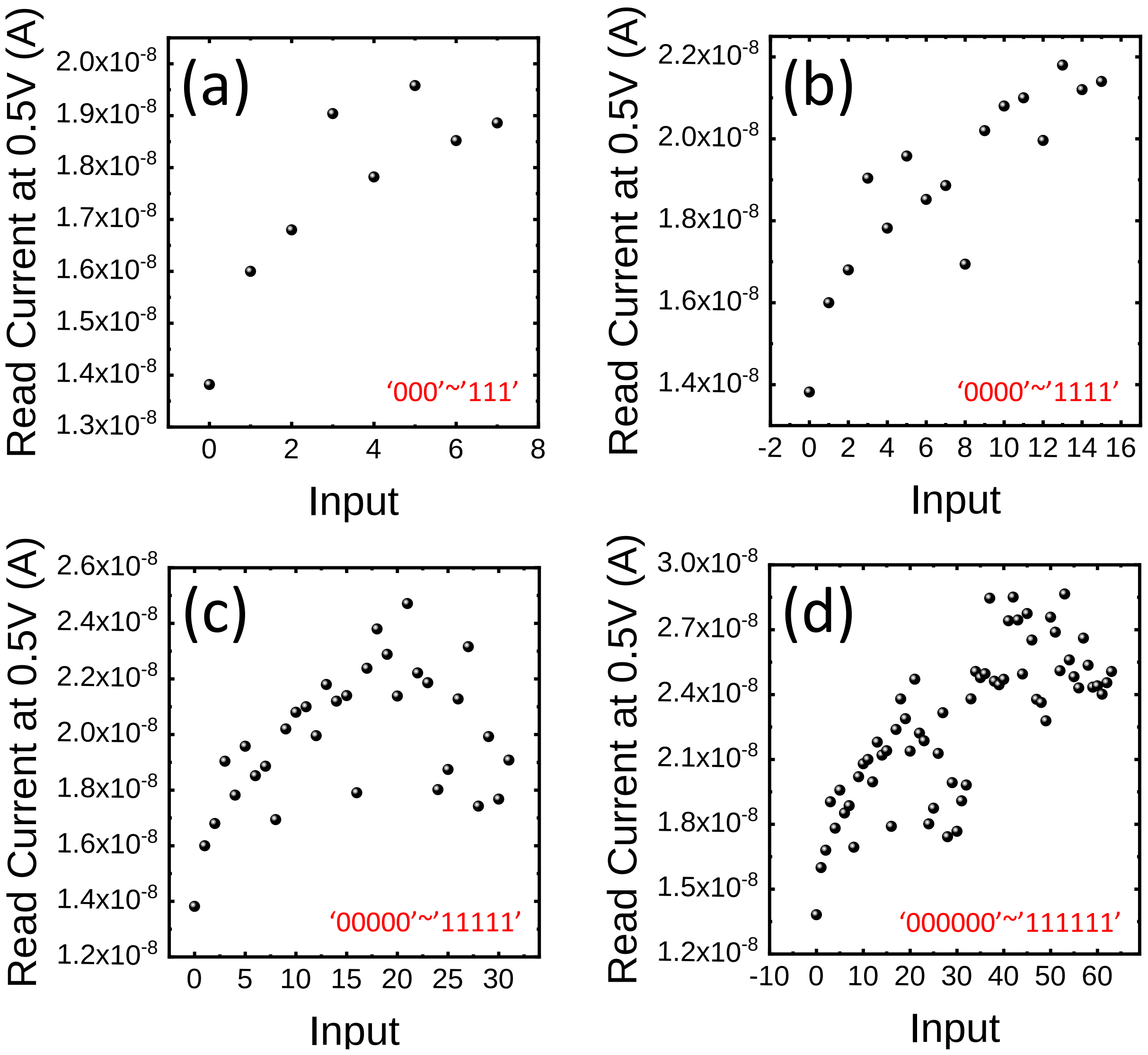

Supplementary Figure S17: The temporal kernel responses were measured while increasing the number of bits processed in the temporal kernel from 3 bits to 6 bits. **a-d)** The temporal kernel responses for the '000~111', '0000~1111', '00000~11111', and '000000~111111' inputs under the conditions of 10 kΩ R_L with a 3.5 V signal pulse height and a 200 ns width, and a 3 V REF pulse height and a 200 ns width. As the number of bits processed in the temporal kernel increased, the separation of the responses to each input deteriorated.

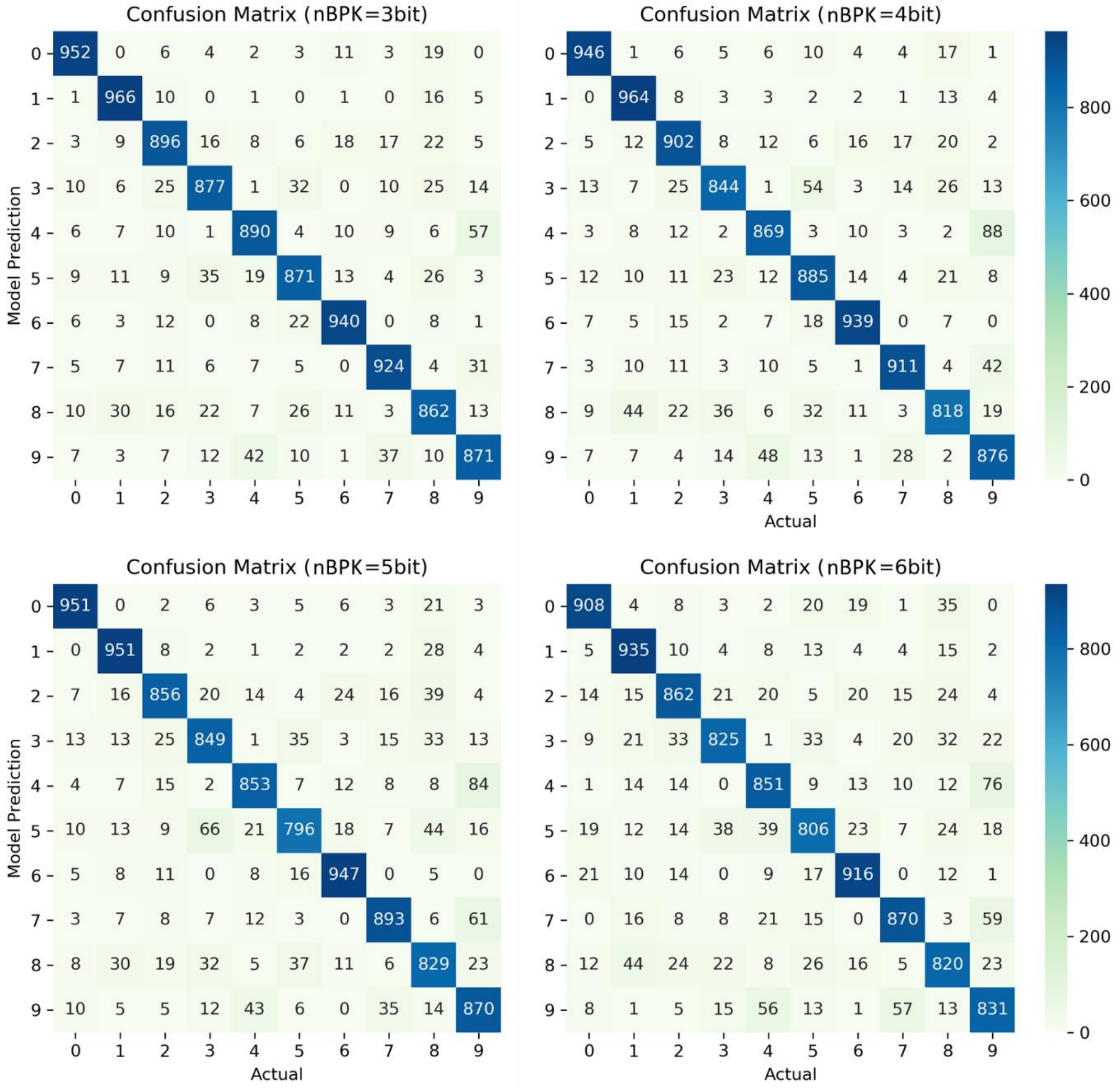

Supplementary Figure S18: The confusion matrices comparing the recognized digit and the desired digit for the MNIST test dataset (4 situations, from the top left: nBPK = 3 bits to the bottom right: nBPK = 6 bits) showing that the number of correct inferences decreased as the nBPK increased

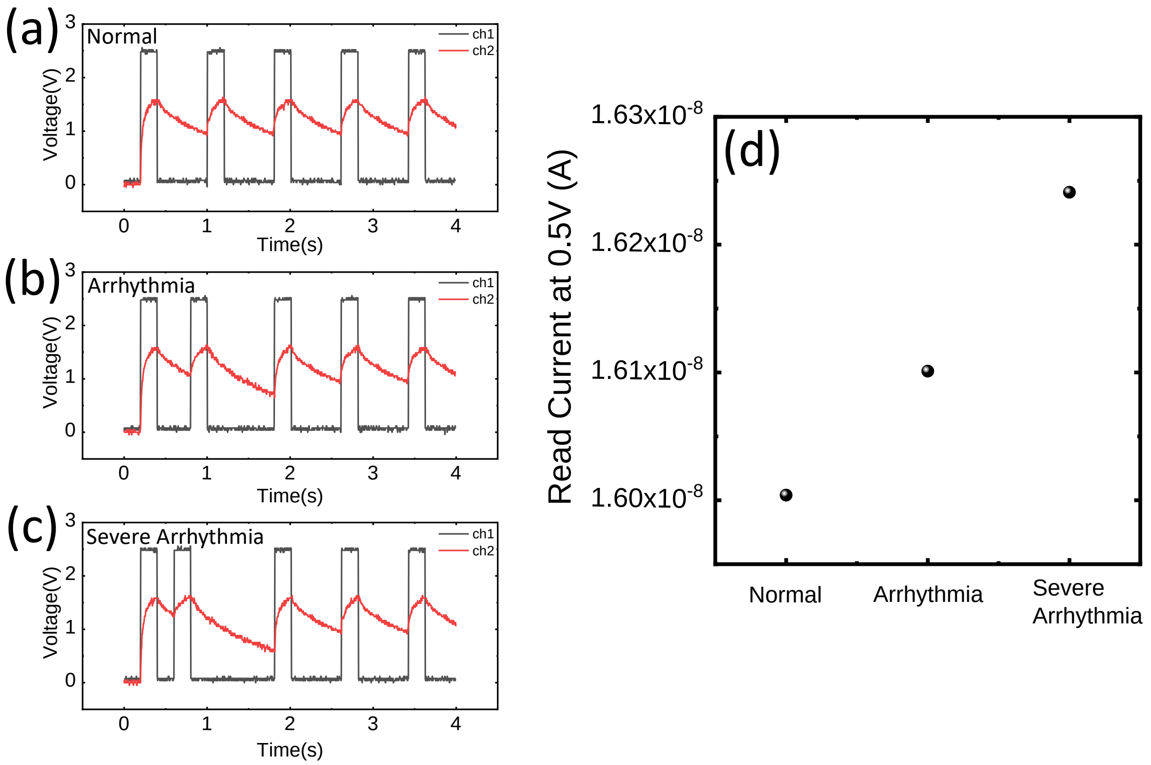

Supplementary Figure S19: The increase in the conductance of the memristor varied according to the degree of arrhythmia. When arrhythmia was severe, SET switching occurred in the memristor due to long discharging. **a-c)** The ECG-based V-t graphs for three cases of normal, arrhythmia, and severe arrhythmia. The electrical signal of the ECG from the heartbeat was converted into a 2.5 V, 200 ms pulse and applied to the memristor. **d)** The read current of the memristor according to the degree of arrhythmia. The more severe the arrhythmia was, the more the memristor conductance increased.

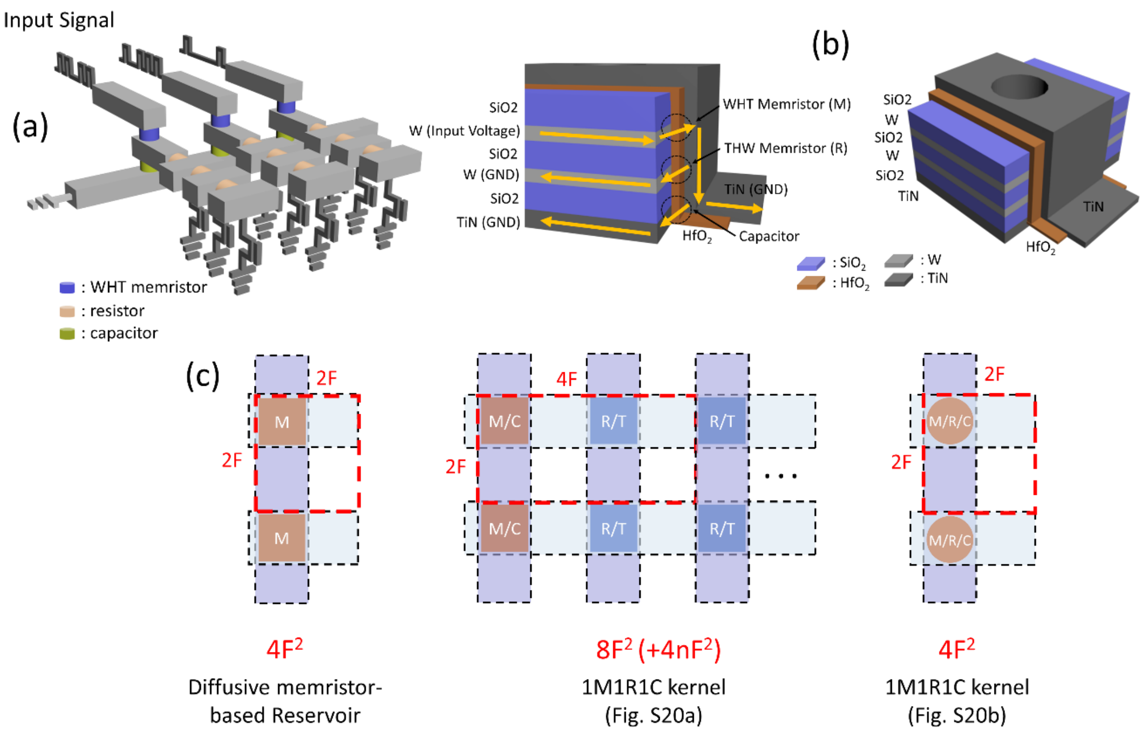

Supplementary Figure S20: The hardware structure needed to create an array of temporal kernels that can adjust the kernel configuration. **a)** A structure in which the resistors are sequentially connected to several metal lines. In this structure, the resistance value of the temporal kernel can be adjusted by selecting several metal lines connected to the resistors. **b)** A structure in which memristors are connected in series to the WHT memristors and parallel to the capacitors (**b left panel**). The 1M1R1C circuit can be implemented in a three-dimensional structure by stacking TiN, W metals in multi-layers and depositing a dielectric layer and top electrode in the hole after hole etching (**b right panel**). In this structure, the resistance of the memristor can be set to the desired resistance value using a method such as the incremental step pulse program (ISPP).[1] **c)** Cell area of the diffusive memristor-based reservoir and 1M1R1C kernel. The diffusive memristor-based reservoir is implemented using a passive array composed of memristors. Therefore, $4F^2$ is required per cell (**c left panel**). If the 1M1R1C kernel is implemented with the structure in **a**, a minimum area of $8F^2$ is required per cell when using a vertical pillar transistor (T)[2], and the area increases by $4F^2$ each time a serial resistor is added

(**c middle panel**). The structure proposed in **b** requires an area of $4F^2$/cell (**c right panel**). This structure does not require an increase of area/cell even with additional elements (R_L, C) other than the memristor through a 3D integration process.

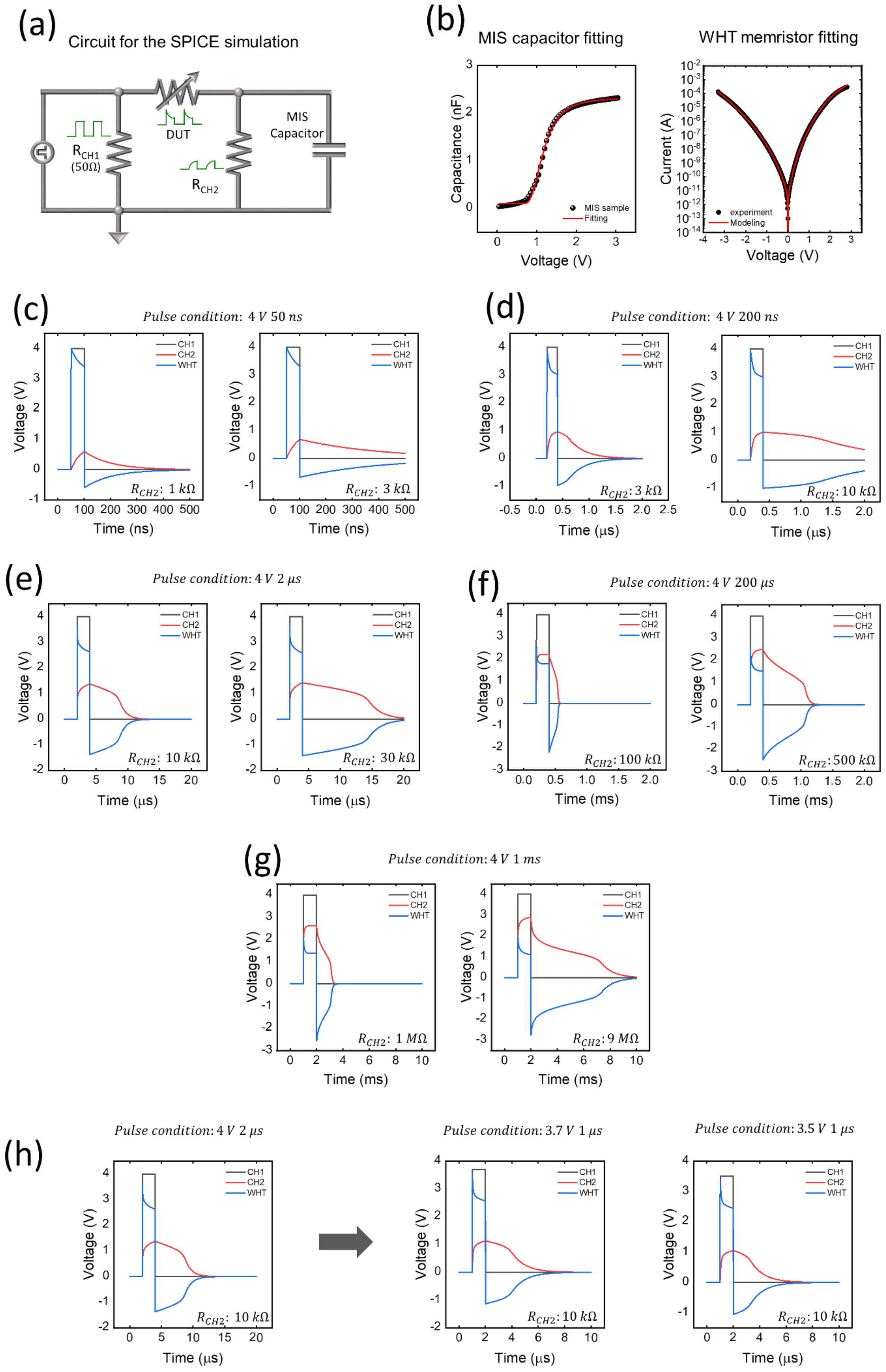
(a)
Circuit for the SPICE simulation
DUT
MIS
Capacitor
RCH1
(50Ω)
RCH2
(b)
MIS capacitor fitting
WHT memristor fitting
Capacitance (nF)
MIS sample
Fitting
Voltage (V)
Current (A)
experiment
Modeling
(c)
Pulse condition: 4 V 50 ns
Voltage (V)
Time (ns)
CH1
CH2
WHT
RCH2: 1 kΩ
RCH2: 3 kΩ
(d)
Pulse condition: 4 V 200 ns
Time (μs)
RCH2: 3 kΩ
RCH2: 10 kΩ
(e)
Pulse condition: 4 V 2 μs
RCH2: 10 kΩ
RCH2: 30 kΩ
(f)
Pulse condition: 4 V 200 μs
Time (ms)
RCH2: 100 kΩ
RCH2: 500 kΩ
(g)
Pulse condition: 4 V 1 ms
RCH2: 1 MΩ
RCH2: 9 MΩ
(h)
Pulse condition: 4 V 2 μs
Pulse condition: 3.7 V 1 μs
Pulse condition: 3.5 V 1 μs
RCH2: 10 kΩ

Supplementary Figure S21: Implementation of various time constants of 1M1R1C kernel using MIS capacitor and WHT memristor (HSPICE simulation). **a**) 1M1R1C circuit used in SPICE simulation. **b**) C-V curve of MIS capacitor and I-V curve of WHT memristor used for simulation and their fitting results (red line). In the simulation, an MIS capacitor (sample device) showing a capacitance of 100 pF ~ 2.2 nF was used, and the WHT memristor fitting result of Supplementary Fig. S16 was used. **c-g**) V-t graphs of the kernel showing fast discharging (left panel) and slow discharging (right panel) characteristics (pulse width 50 ns ~ 1 ms). **h**) V-t graphs in the kernel condition of 4 ~ 3.5 V pulse height, 2 ~ 1 μs pulse width, and 10 kΩ R_{CH2}. **h** shows the effect of changing pulse height on the capacitance of the TK system.

Supplementary Tables

Supplementary Table 1: The frequency of the appearance of inputs in the preprocessed MNIST dataset, in which '0000' appeared overwhelmingly, followed by the inputs '1111', '1000', '0011', '0001', '1100', '0111' and '1110' in the table (Due to the nature of the picture, the pixels were continuously blanked or filled in most cases. Therefore, inputs with consecutive high or low signals mainly appeared, and there were a few inputs with alternating high and low signals such as '1010' and '0101'.)

Inputs	# of Inputs	Percentage [%]
0000	9,078,931	77.2017
0001	355,307	3.0213
0010	24,104	0.2049
0011	356,362	3.0302
0100	23,171	0.1970
0101	806	0.0068
0110	105,121	0.8938
0111	276,838	2.3540
1000	364,691	3.1011
1001	11,919	0.1013
1010	959	0.0081
1011	8,580	0.0729
1100	346,940	2.9501
1101	9,044	0.0769
1110	262,350	2.2308
1111	534,877	4.5482
Total	11,760,000	100

Supplementary Table 2: Results of MNIST recognition using various kernel combinations. For the recognition, kernel conditions of Figs.3a, b, and f of main text were used. A combination of 'Fig.3a+Fig.3f' showed an accuracy of 91.8%. For a 196x10 input vector, two kernels processed the input, and a 392x10 readout layer was used (588x10 readout for the 3 kernels). On the other hand, when the pulse width was modified without changing the conditions R_L, C, and pulse height in the condition of Fig. 3f, an accuracy of 92.4 % was obtained in the combination of '200ns+2μs+5μs'. By combining various kernels or changing pulse conditions for the same kernel machine, the imperfections of one kernel could be compensated for by another kernel, and the accuracy could be improved.

Kernel	Accuracy	Input vector	Readout Layer
Fig.3a + Fig.3b	90.7%	196x10	392x10
Fig.3a + Fig.3f	91.8%	196x10	392x10
Fig.3b + Fig.3f	91.7%	196x10	392x10
Fig.3a + Fig.3b + Fig.3f	91.7%	196x10	588x10
Kernel	Accuracy	Input vector	Readout Layer
200 ns + 2 us	91.4%	196x10	392x10
200 ns + 5 us	91.6%	196x10	392x10
2 us + 5 us	91.5%	196x10	392x10
200 ns + 2 us + 5 us	92.4%	196x10	588x10

Supplementary Table 3: The accuracy when cycle to cycle variation, cell to cell variation, and both are considered (kernel condition of Fig. 3f of main text was used). Each variation was calculated based on variation measurement results in Supplementary Fig S1. Up to 1 sigma of each variation was considered, and when both cycle to cycle and cell to cell varaition were included in the simulation, the accuracy decreased by 0.5 %.

Variation	Accuracy
no variation	90.1%
cycle to cycle	89.7%
cell to cell	90.0%
cycle to cycle + cell to cell	89.6%

Supplementary Table 4: Results of MNIST recognition using two-layer FCN for the readout layer of TK system. The table shows the number of training parameters used in each two-layer FCN and the accuracy of the TK system (nBPK = 4). When 196x38x10 FCN was used, 7,828 training parameters were used, and the TK system accuracy was 95.1 %.

Readout layer	Training parameters	nBPK	Accuracy
196 x 38 x 10	7,828	4	95.1 %
196 x 50 x 10	10,300	4	95.5 %
196 x 110 x 10	22,660	4	96.1 %
196 x 200 x 10	41,200	4	96.5 %

Supplementary Note 1. Area-type electronic switching memristor

The switching mechanism of the W/HfO_2/TiN device used in the experiment was analyzed based on various experiments. First, the area dependency of the device conductance, shown in Supplementary Figs. S4c,d, shows that this device is not a filamentary type. When the conducting filament is formed, the conductance is independent of the device area because the current flows locally.[3] Second, the temperature dependence of the current shown in Supplementary Figs. S4a,b shows that this device is not metallic in the LRS. When the filamentary type device is in the LRS, a metallic conducting filament is formed, and the current decreases as the temperature rises.[4] In this device, the current increases as the temperature increases. Lastly, in Supplementary Fig. S5, the activation energy in various conductance states was calculated, and reasonable values that corresponded to the trap level of the HfO_2[5] were calculated. These support the operation of the device based on the trap-assisted switching mechanism.

Supplementary Note 2. Comparison of how the temporal kernel responded to the input according to various parameter changes (Fig. 3a-e)

Figure 3b shows that R_L had two main effects on the kernel dynamics. First, when R_L was changed, the distributed voltage varied. When the voltage distributed to the memristor increased, the separation of the inputs, including the consecutive ‘1’, increased. Typically, the separability for the inputs ‘0000’ (= 0), ‘0001’ (= 1), ‘0011’ (= 3), ‘0111’ (= 7), and ‘1111’ (= 15) increased. This is because when the R_L was small, even with the distributed voltage, considerable SET switching occurred (Supplementary Fig. S12). The second effect is the change in the discharging rate. Too fast or too slow discharging reduced the kernel separability. For example, the effect of the interval between spikes can be neglected by too fast discharging. In Fig. 2b, the kernel responses for the inputs ‘1000’, ‘1001’, and ‘1011’ are not identified separately. Supplementary Fig. S8 shows that the second spike was not significantly affected

by the interval length due to the fast discharging. As the discharge rate increased, the input that caused the maximum conductance changed from '1000' to '1010' (Supplementary Fig. S13).

Figure 3c shows the TK responses when the pulse width was increased to 200 μs from the conditions in Fig. 3a. As the pulse width increased, the amount of discharging during one low signal increased, so its effect was similar to that when R_L decreased, but the magnitude of the voltage distributed to the memristor did not change, so the effect of the capacitor discharging appeared more clearly. Figure 3c shows that when R_L was large, set switching occurred according to the spike rather than according to the voltage distributed to the memristor. When '0000' ~ '1111' inputs were applied to this system, one to three net spikes were generated. Figure 3c shows that the TK response was divided into only three states by the number of net spikes.

Figure 3d shows the TK response when the pulse height was changed to 3.5 V. In this case, the responses to the inputs were not exactly the same because the size of the spike and the size of the divided voltage differed. However, it can be seen that the characteristic itself did not significantly differ from that in Fig. 3a.

Figure 3e shows the TK responses when the signal pulse height was kept at 4 V but the REF pulse height was lowered to 3 V. In this case, since the '0000' input received only REF pulses, the SET switching was much less than in the other cases, and the TK response to the '0000' input was reliably separated. Also, when the REF pulse height differed from the signal pulse height, the inputs with two spikes, such as '0010' and '0101', were separated from each other. In '0010', the second spike was generated by the REF pulse, and in '0101', the second spike was generated by the signal pulse. In the case of '0010', the SET switching was much less, and the two inputs were separated. Therefore, unlike in Fig. 3a, where it is difficult to distinguish the inputs, in Fig. 3d, it can be seen that the '0010' and '0101' inputs are well separated.

Supplementary References

1. Suh, K. D. *et al.* A 3.3 V 32 Mb NAND Flash Memory with Incremental Step Pulse Programming Scheme. *IEEE J. Solid-State Circuits* (1995) doi:10.1109/4.475701.
2. Song, K. W. *et al.* A 31 ns random cycle VCAT-based $4F^2$ DRAM with manufacturability and enhanced cell efficiency. in *IEEE Journal of Solid-State Circuits* (2010). doi:10.1109/JSSC.2010.2040229.
3. Kim, K. M., Choi, B. J., Shin, Y. C., Choi, S. & Hwang, C. S. Anode-interface localized filamentary mechanism in resistive switching of TiO_2 thin films. *Appl. Phys. Lett.* (2007) doi:10.1063/1.2749846.
4. Privitera, S. *et al.* Microscopy study of the conductive filament in HfO_2 resistive switching memory devices. *Microelectron. Eng.* (2013) doi:10.1016/j.mee.2013.03.145.
5. Driussi, F., Spiga, S., Lamperti, A., Congedo, G. & Gambi, A. Simulation study of the trapping properties of HfO_2-based charge-trap memory cells. *IEEE Trans. Electron Devices* **61**, 2056–2063 (2014).
